# Dietary flavonoids form supramolecular assemblies, alter biochemistry, and enhance cell resilience

**DOI:** 10.3389/fnut.2025.1649867

**Published:** 2025-09-02

**Authors:** Charles B. Reilly, Sylvie G. Bernier, Sanjid Shahriar, Viktor Horvath, Michael Lewandowski, Emilia Javorsky, Bogdan Budnik, Donald E. Ingber

**Affiliations:** ^1^Wyss Institute for Biologically Inspired Engineering at Harvard University, Boston, MA, United States; ^2^Harvard John A. Paulson School of Engineering and Applied Sciences, Harvard University, Cambridge, MA, United States; ^3^Vascular Biology Program and Department of Surgery, Harvard Medical School and Boston Children’s Hospital, Boston, MA, United States

**Keywords:** supramolecular assembly, molecular dynamic simulation (MDS), polyphenols, flavonoids, radiation

## Abstract

Flavonoids, a diverse class of polyphenols found in many plant-based foods, are increasingly recognized for their health benefits, including antioxidant and anti-inflammatory activities. Recent evidence links higher dietary flavonoid diversity with reduced all-cause mortality and improved outcomes in chronic diseases. Yet, due to concerns about non-specific binding, their exclusion from drug development pipelines has limited deeper mechanistic understanding. Here, we show how flavonoids may promote cellular resilience by forming supramolecular assemblies that interact with proteins. Using molecular dynamics simulations (MDS) and *in vitro* assays, we found that different flavonoids self-assemble into ordered structures that influence protein structural dynamics and fiber formation. These structures have differential effects on enzyme activity and cell viability under stress. The ability to undergo supramolecular assembly may be important as flavonoids protect human cells against ultraviolet radiation-induced damage through a non-antioxidant mechanism. These findings suggest that supramolecular assembly and structural heterogeneity of flavonoids may underlie their diverse bioactivities and help to explain how the diversity of dietary flavonoids can support adaptive changes in cellular biochemistry, enhance resilience to environmental stressors, and improve human health.

## Introduction

Flavonoids are a diverse class of polyphenols widely found in fruits, vegetables, nuts, seeds, and other plant-based foods. These compounds are associated with a broad range of health benefits, including antioxidant, anti-inflammatory, and cytoprotective effects (1–4). Importantly, a recent large-scale population study showed that greater dietary diversity of flavonoid intake is associated with reduced all-cause mortality and decreased risk of major chronic diseases (5). These studies suggest that diversity in dietary flavonoids—not just quantity—may be critical for promoting long-term physiological resilience.

However, the mechanism through which their activity is achieved is not well characterized, and their use has not been explored in more conventional therapeutic development programs. In fact, flavonoids, such as quercetin (6–10), are commonly excluded from high-throughput drug screening programs because they cause pan-assay interference (PAINS), leading to false positive results (11). This activity is assumed to be due to the non-specific binding of flavonoid molecules to proteins or other biological targets. Use of PAINS filters (12) in high-throughput screening has been critical in reducing false positive hits and improving overall drug screening efficiency and accuracy. This efficiency is important in conventional drug design, where the desired therapeutic behavior is based on a drug or drug-like molecule fitting into a specific binding site on a target protein.

Given their known health benefits, high oral tolerability, and the recent finding that diverse flavonoid intake reduces all-cause mortality (5), flavonoids could represent overlooked therapeutic candidates. They are often overlooked because they are excluded from conventional drug discovery pipelines. Therefore, we carried out exploratory biochemical studies and molecular dynamics simulations (MDS) to better understand how flavonoids might influence the biochemical activities of multiple unrelated enzymes. Surprisingly, we discovered that flavonoids slow molecular biochemistry through higher-order self-assembly into multimolecular structures that physically impinge on the molecular motion of enzymatic proteins.

The heterogeneity of flavonoid structures also appears to be a central feature of both their bioactivity and cellular responses. For example, in the context of aging and senescence, flavonoids have been proposed to modulate stress pathways through diverse and context-dependent interactions (13). We explore this hypothesis by examining how supramolecular assembly may introduce structural variability across biomolecular systems, potentially contributing to adaptive regulation under environmental stress. Rather than seeking single-target specificity, this approach considers dietary compounds as dynamic modulators within heterogeneous biological networks.

## Results

**Figure 1:**
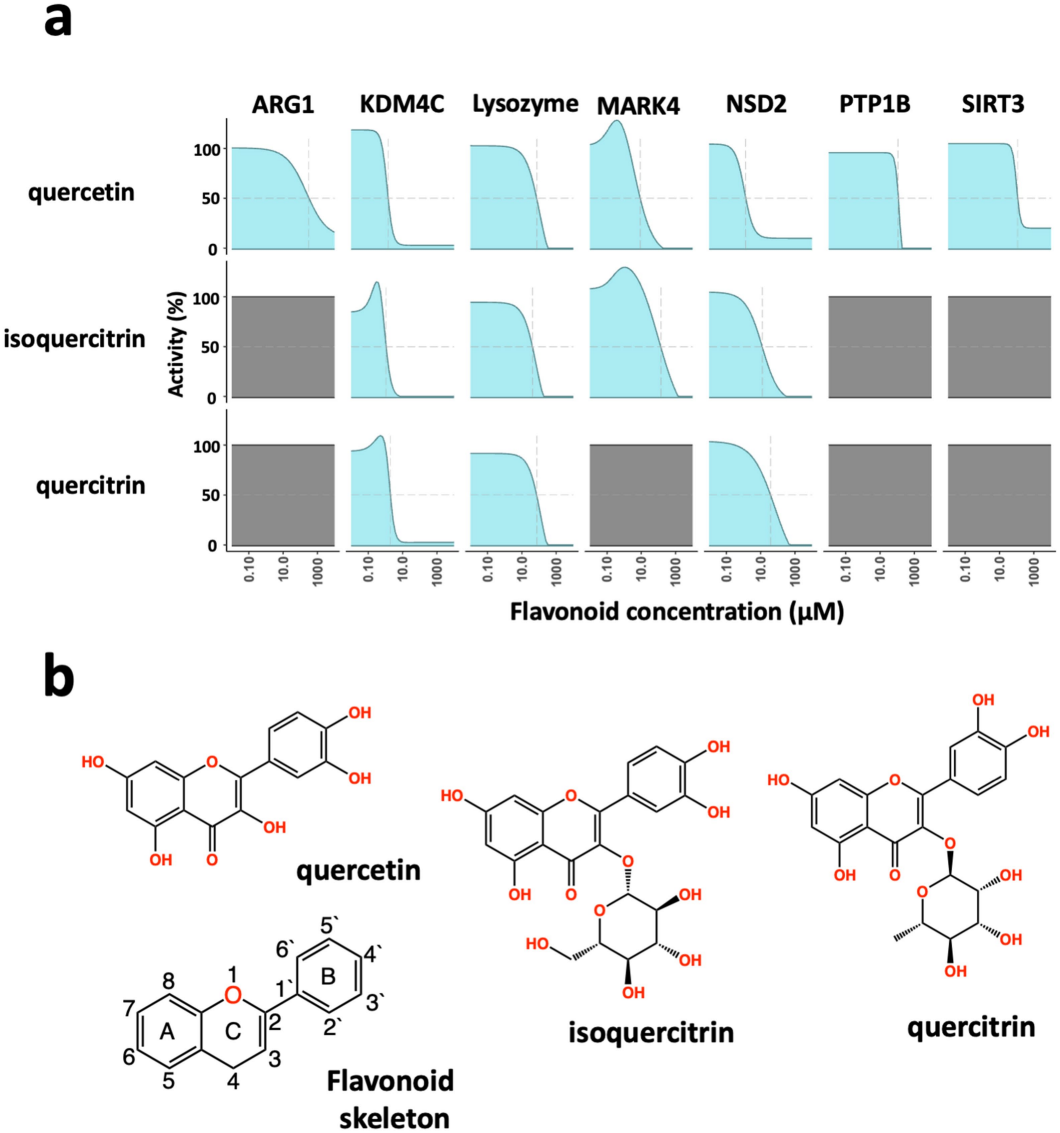
Broad-spectrum inhibition of enzymatic activity by flavonoids. **(a)** Dose-dependent inhibition of enzyme activity. In biochemical assays, the flavonoids quercetin, isoquercitrin, and quercitrin show different enzyme inhibition of Arginase 1 (ARG1), Lysine demethylase 4C (KDM4C), lysozyme, MAP/microtubule affinity-regulating kinase 4 (MARK4), Histone-Lysine N-methyltransferase (NSD2), Tyrosine-protein phosphatase non-receptor type 1 (PTP1B), and Siturin-3 (SIRT3). Quercitin showed concentration-dependent inhibition of all seven enzymes; all three flavonoids blocked the enzymes KDM4C, Lysozyme, and NSD2; while quercitrin performed similarly to isoquercitrin, it did not inhibit the MARK4 enzyme. **(b)** Chemical structures of the flavonoids, quercetin, isoquercitrin, and quercitrin, as well as the core flavonoid skeleton.

First, we carried out experimental studies to investigate the impact of 58 different flavonoids on biochemical activities using seven different enzyme assays, including Arginase 1 (ARG1), Lysine demethylase 4C (KDM4C), Lysozyme, MAP/microtubule affinity-regulating kinase 4 (MARK4), Histone-Lysine N-methyltransferase (NSD2), Tyrosine-protein phosphatase non-receptor type 1 (PTP1B), and Siturin-3 (SIRT3; Supplementary Table 1). Results from enzyme activity analysis revealed that subtle variations in flavonoid structure led to varying specificity in enzyme activity inhibition (Supplementary Table 2). We focused on 3 of the 58 flavonoids that showed distinctly different enzyme specificities (quercetin, isoquercitrin, and quercitrin) to gain further insight into the underlying mechanism responsible for their observed inhibitory effects. Quercetin, the smallest compound with a core flavonoid structure, inhibited all tested enzymes. At the same time, isoquercitrin and quercitrin, which share the same core flavonoid structure as quercetin, demonstrated varied levels of inhibition (Figure 1a). The primary structural difference between quercetin and the other two flavonoids is the presence of a glycosidic group instead of a hydroxyl group in the number 3 position of the C-ring—glucose in isoquercitrin and rhamnose in quercitrin (Figure 1b). These glycosidic groups differ not only in composition but also in the position of their hydroxyl groups.

To gain mechanistic insight into how these subtle structural variations in flavonoids may lead to different impacts on enzyme inhibition, we conducted MDS on two enzymes: MARK4 and PTP1B, which showed varied activity in the presence of the three highlighted flavonoids. Quercetin inhibited both enzymes, quercitrin did not affect either enzyme, and isoquercitrin inhibited MARK4, but not PTP1B. The starting structures of enzymes used in the simulations were generated using AlphaFold (14), and they both exhibited disordered regions with low structural integrity. Simulations were performed with a consistent stoichiometry (1:1 ratio by mass of enzyme protein to quercetin).

**Figure 2:**
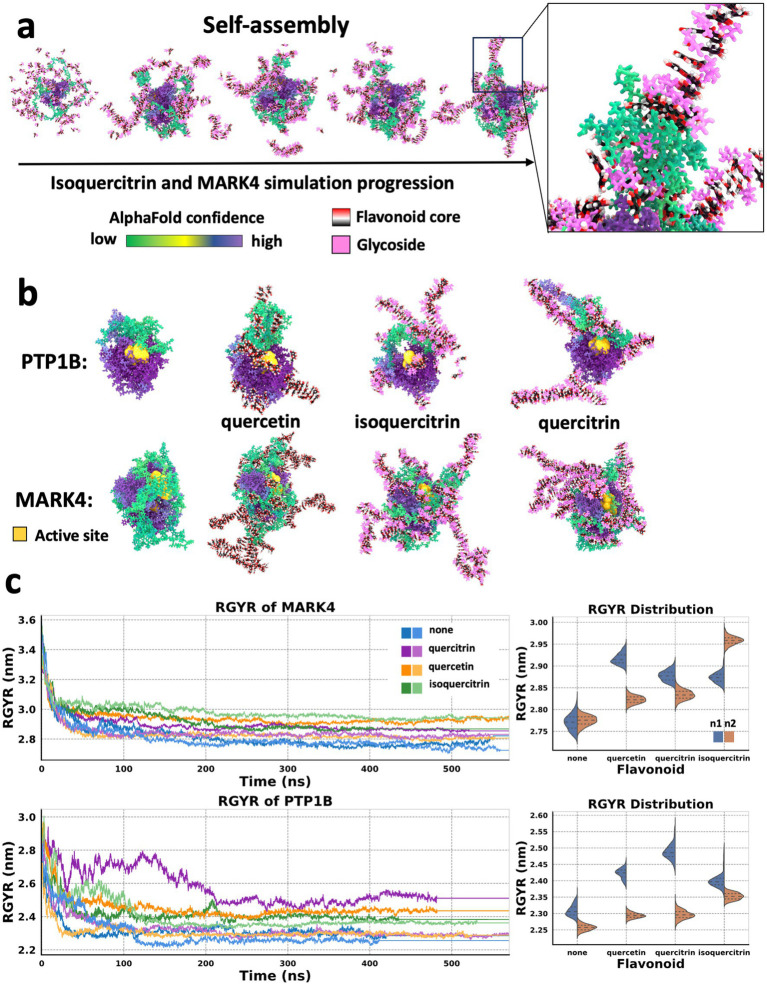
Flavonoids form higher-ordered structures that influence the molecular dynamics of proteins. **(a)** States taken every 25 ns from an MD simulation of a MARK4 enzyme molecule in the presence of isoquercitrin over the course of a 100 ns MD simulation. Self-assembly of a supramolecular structure involving the flavonoids and the protein. The proteins are colored based on AlphaFold confidence score. The flavonoid cores are rendered based on element, and the flavonoid’s glycosidic moiety is pink. **(b)** States taken after 100 ns MD simulation of PTP1B and MARK4 enzymes in the presence of flavonoids, with the active site enlarged and in yellow. (left to right) Protein alone, protein and quercetin, protein and isoquercitrin, and protein and quercitrin. **(c)** Radius of gyration (RGYR) analysis of simulations in panel **b** run in duplicate, and violin plots of the RGYR values from 200 to 400 ns with dotted lines indicating quartiles and color simulation run. The violin plots show the variance in RGYR between the replicates for a trajectory range from 200 to 400 ns for each flavonoid-protein system. This trajectory region was selected to ensure the systems were well equilibrated.

Each system was simulated for over 400 ns in duplicate. Notably, after approximately 100 ns, the simulations indicated that the flavonoids self-assembled into stable, highly ordered supramolecular structures that remained intact throughout the duration of the simulation (Figure 2a). These flavonoid assemblies interacted uniquely with the enzymes, with variations dependent on the specific flavonoid type. Interestingly, the variance between duplicate simulations for the flavonoid-containing systems was greater than the variance observed in duplicates of the control (protein-only) systems. This suggests that the presence of flavonoids introduces additional structural variability that is sensitive to the specific flavonoid. Such structural heterogeneity may reflect a range of physiologically relevant interaction modes. Notably, these interactions influenced the conformational states of the disordered enzyme protein regions, and the supramolecular flavonoid structures formed in these protein regions varied. With both MARK4 and PTP1B enzymes, the supramolecular flavonoid structures impacted the protein backbone structure near the enzyme’s active site (Figure 2b). These subsequent structural changes to the disordered protein regions likely influence the substrate orientation and its access to the enzyme’s active site, suggesting a potential mechanism for enzyme regulation.

Next, we investigated whether flavonoid supramolecular assemblies alter the overall structural dynamics of the two enzymes. To do this, we performed a radius of gyration (RGYR) analysis, which indicates the overall compactness of the protein. The flavonoids all increased the RGYR of both proteins relative to the proteins alone (Figure 2c). The control duplicates also had similar RGYR after 300 ns. Still, there was more variation between the duplicates when flavonoids were present, suggesting that flavonoids increase the structural heterogeneity of these proteins. Using a Levene test, we found that this variance in the presence of flavonoid was significant in all cases (*p* < 0.005) compared to the protein alone. However, PTP1B with no flavonoid still had RGYR variation, while PTP1B protein with isoquercitrin had an increase in RGYR but with less variance than the other flavonoids.

This heterogeneity in each protein/flavonoid assembly may contribute to altered enzyme activity *in vitro.* This is in line with past studies by ourselves and others (15–17). These studies have shown that, using single-molecule enzymology, a homogenous population of enzymes can display heterogeneous activities when individual enzyme molecules are measured. This activity distribution can be tuned with environmental parameters, such as heat and pH. Thus, multiple supramolecular assemblies may form within a population of enzyme molecules with the same protein-flavonoid composition. This heterogeneity is not observable in conventional biochemical assays, such as those used in this study, which measure only bulk enzymatic activity.

Another way enzyme activity could be modulated by the formation of assemblies may be through the sequestration of enzyme substrates within the supramolecular structures. We carried out simulations with adenosine triphosphate (ATP) to see if the substrates of MARK4 kinase are sequestered in supramolecular complexes with the flavonoids, which would also contribute to the modulation of enzyme activity. PTP1B is a phosphatase, so the primary substrate involved with the enzyme is the protein it is dephosphorylating. In the simulations, we also included adenosine diphosphate (ADP) as the sequestering of the enzyme product could also influence enzyme activity.

When we performed simulations with 10 flavonoids, five ATP, and five ADP molecules, we found that the nucleotides were not sequestered into the flavonoid supramolecular structure. The 100 ns simulations were repeated in triplicate for quercetin, quercitrin, and isoquercitrin. Each time, the flavonoids formed structures, and the nucleotides did, too, but the structures that formed were not interconnected. Final states of one replicate for each flavonoid (which are shown in Supplementary Figure 1a; Supplementary Videos 1a–c) show the assembly process over 100 ns for all nine simulations. Although the final structures were not interconnected during the assembly process, flavonoids and nucleotides formed transient interactions that can be observed in the videos. The formation of ATP assemblies is consistent with other studies that have reported ATP aggregation and the impact this can have on reducing protein aggregation in cells (18, 19).

Interestingly, the flavonoid supramolecular assemblies formed fibers. Fiber formation has also been observed in ensembles of intrinsically disordered proteins (IDPs) (20), which can protect cells against environmental stressors. Fiber formation is crucial for proteins that make up the cytoskeleton via non-covalent assemblies, which structurally regulate cellular physiology through the transmission of mechanical forces (21, 22). Given their length, it is possible that these long self-assembled flavonoid structures could also influence protein–protein interactions, extending their potential impact beyond the modulation of enzyme action. The altered degrees of freedom the fibers introduce may also impact enzyme activity.

**Figure 3:**
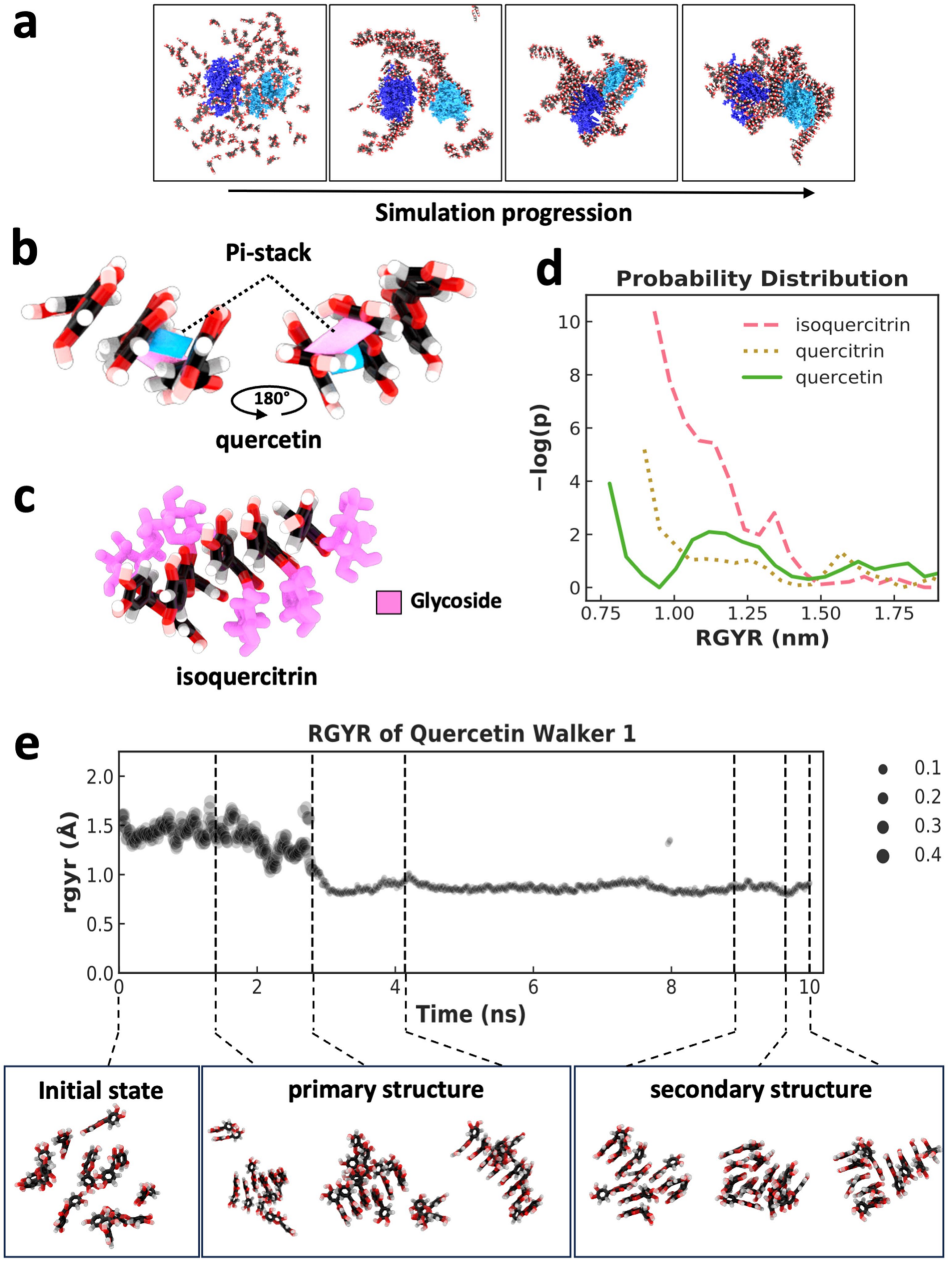
Supramolecular assembly formation and weighted ensemble simulations of flavonoids. **(a)** States taken every 100 ns across a 400 ns MDS of two lysozyme molecules (dark blue and light blue) and 128 quercetin molecules, indicating the self-assembly of a supramolecular structure comprised of flavonoid and two initially separated protein molecules. **(b)** Pi-stacking (shown in pink and blue between two molecules) leading to the assembly of three quercetin molecules. **(C)** assembly of five isoquercitrin molecules via pi-stacking of a flavonoid core. Glycosidic groups (pink) are positioned on the outside of the assembly, forming a “sugar backbone.” **(d)** Indicator of assembly size and compactness using a weighted probability distribution of supramolecular structures with different RGYR. The plot represents distribution across 500 ns of simulation using 50 walkers in a weighted ensemble simulation with an RGYR distance function. **(e)** RGYR trace and weighting over the 10 ns trajectory of a single walker (top) and a selection of quercetin states taken from across the trajectory (bottom) with indicated RGYR. Primary and secondary structure examples are shown along with initialization states that lack a supramolecular structure.

To investigate the assembly of flavonoid supramolecular structures in the context of multiple protein molecules, we conducted MDS of flavonoid self-assembly in the presence of two Lysozyme molecules. During simulation, the enzyme molecules became interconnected by the flavonoid assembly, forming a unified structure (Figure 3a; Supplementary Figure 2). Analysis of the radius of gyration (RGYR) confirmed that the flavonoid assembly resulted in physical interconnections between neighboring Lysozyme molecules, as the entire unit of two protein molecules and bound flavonoid structures moved together as a single mass, which was indicated by a reduction in the variation of RGYR values (Supplementary Figure 2a). Moreover, MDS analysis and visualization of force distributions within the self-assembled structures (Supplementary Figures 2b,c) revealed that forces could be transmitted between the two protein molecules via the flavonoid assemblies. This mechanotransduction mechanism via allostery between different molecules is a common feature observed in protein complexes that undergo conformational shifts to fulfill their functional roles (21–24).

Through visual inspection by MDS, the supramolecular flavonoid structures that formed underwent self-assembly as a result of pi-stacking mediated by the specific flavonoid’s polyphenol substructure. MDS visualization of quercetin undergoing this process shows how the flavonoid core can form two pi-stacking interactions at two angles (Figure 3b). Pi-stacking of the flavonoid core strikingly resembles the stacking of base pairs within nucleic acids. The glycosidic groups in quercitrin and isoquercitrin also seemed to influence the assembly process (Figure 3c). For example, when the glycoside sugar is present as it is in isoquercitrin, the supramolecular structure forms so that the sugar is on the outside of the pi-stacking, which leads to the formation of a sugar backbone and the whole structure takes on a helical form, again like in nucleic acids (Figure 3c) (25).

To quantitatively assess whether the glycosidic groups promoted or hindered the formation of ordered structures, weighted ensemble simulations (WES) were conducted to explore the associated free energy landscape. A WES system was set up for each of the three flavonoids with 10 molecules in explicit solvent, employing 50 walkers and a total simulation time of 500 ns using a resampler to utilize the RGYR (which measures the average distance of atoms from the center of mass or compactness) as a distance function. A free energy profile was calculated by normalizing the weighted distribution of RGYR values, followed by transformation using -ln(p) to obtain a free energy-like value (26), and then each flavonoid’s resulting −log(*p*) probability distribution was calculated (Figure 3d). The RGYR of WES initialization (when no self-assembly has occurred) was ~1.5 nm, and assembly caused the RGYR to decrease. The plot in Figure 3d indicates significant separation in the probability distribution of the different flavonoids forming structures smaller than 1.5 nm, with quercetin showing the highest probability of developing the most compact structures (smaller RGYR) as opposed to the flavonoids with glycosidic groups.

The weighted RGYR of a single representative walker lineage for a 10 ns trajectory for the quercetin system is shown in Figure 3e. Seven states across the trajectory are shown (Figure 3e bottom) to indicate the transition from a state with RGYR ~1.5 nm to a state with < 1 nm and the formation of primary and secondary structures throughout the trajectory. In addition to parallel pi-stacking, there are other forms (27) of interactions involving pi systems, and this can lead to altered geometry, which we observed in the form of primary and secondary structural forms facilitated by pi-stacking interactions and hydrogen bonding of hydroxyl groups previously identified through visualization. The primary structure of the flavonoid fiber has one stack of molecules connected by pi-stacking, and a secondary structure leads to additional dimensionality in the supramolecular structure. This secondary structure is formed when a second primary structure branches out perpendicular or parallel to the first. This secondary structure formation is facilitated via a branching pi-stacks and/or hydrogen bonding. Interestingly, the glycosidic groups can significantly influence the orientation of the secondary structure due to glycosidic backbone formation.

In our study of flavonoid behavior, the presence of additional hydroxyl groups within the glycosides and the flexibility associated with these sugars compared to the polyphenol core seem to decrease the probability of forming the most compact structures with the smallest RGYR. This reduced likelihood to form compact structures may indicate an ability to develop more diverse, higher-dimensional structures that are larger and less compact. This distinction is notable when more complex flavonoids are compared to quercetin, which only has hydroxyl groups directly attached to the polyphenol core. The ability to form more complex structures would suggest a potential for building elaborate hierarchical arrangements with branching patterns, resulting in improved stabilization effects on both single and multiple macromolecules. Moreover, the flexible capacity of these flavonoids to form three-dimensional structures can likely facilitate adaptive interactions with other biomolecules, such as proteins, as we observed (Figure 2). Our results are consistent with the possibility that the increased probability of developing flavonoid-mediated structures with higher dimensionality may contribute to enhanced stabilizing effects. Our studies suggest that flavonoid glycosidic moieties influence the assembly process, the diversity of fully assembled molecular states, and the formation of secondary structures.

Notably, we observed increased variance in structural dynamics (RGYR) when flavonoids were present. Rather than treating this as noise, we interpret this heterogeneity as a biologically meaningful feature of flavonoid–protein interactions. Such heterogeneity may enable diverse structural outcomes within a population of enzymes, supporting flexible biochemical responses under stress. This aligns with emerging views in nutritional science that dietary diversity contributes to adaptive physiological regulation and improved human health (5). We emphasize that these simulations are qualitative and were intended to guide hypothesis generation, rather than provide mechanistic proof. While they offer insight into potential supramolecular organization and protein interactions, further work is required to rigorously quantify these effects using advanced modeling and experimental validation.

**Figure 4:**
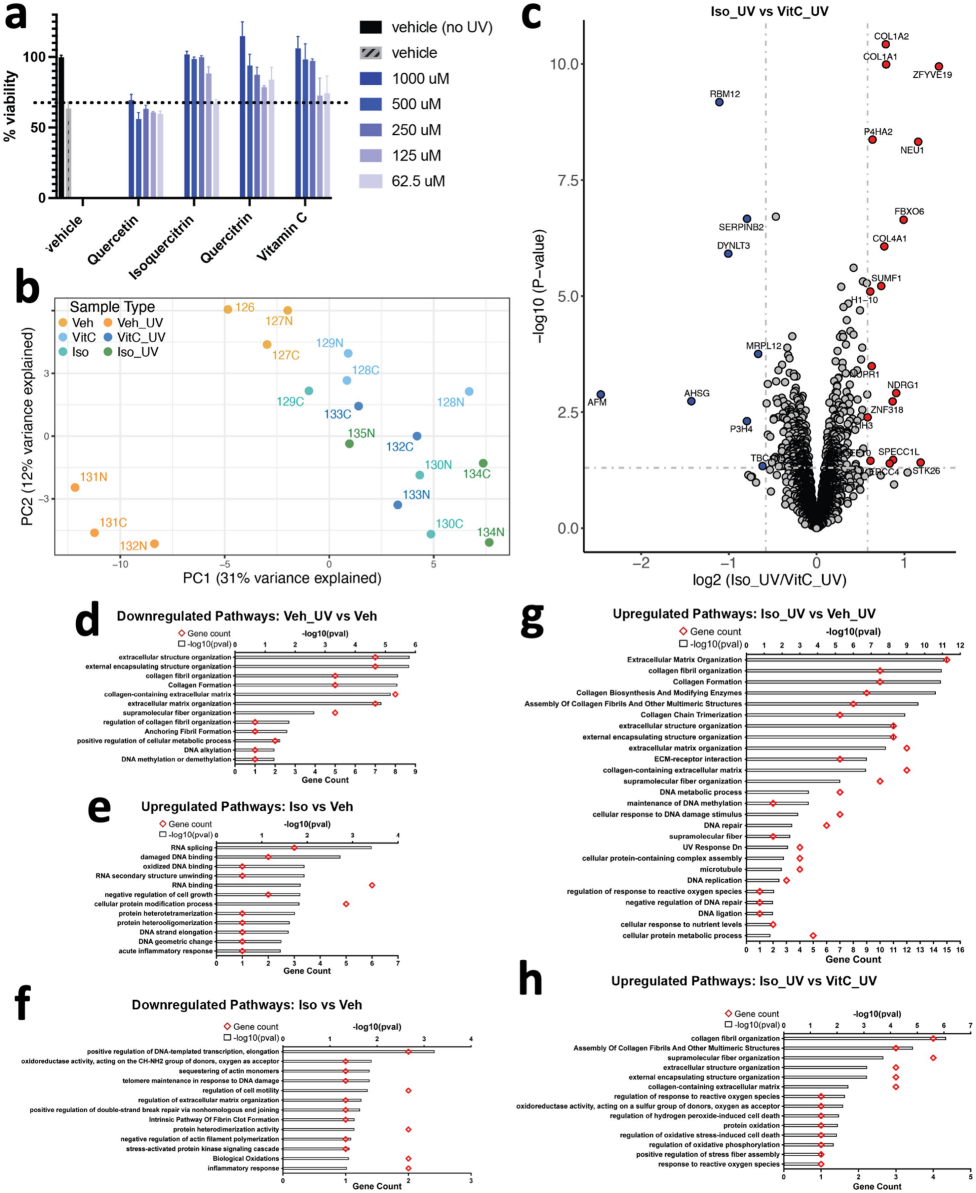
Flavonoids protect human dermal fibroblasts against ultraviolet radiation-induced cell death in a dose-dependent manner. **(a)** Human fibroblasts were pretreated with the indicated concentration of compounds for 1 h prior to ultraviolet radiation exposure. Cells were cultured in complete medium for an additional 24 h, and cell viability was tested by CellTiter-Glo assay. Results are expressed as means ± SEM of three independent experiments. **(b)** Principal Component Analysis (PCA) plot of proteomics data. Each data point represents a sample labeled with TMT (Tandem Mass Tag) and is colored according to its sample type. **(c)** Volcano plot of proteins differentially expressed between fibroblasts exposed to UV light and treated with isoquercitrin or Vitamin C. Gray dotted horizontal and vertical lines denote a *p*-value cutoff of 0.05 and log2fc cutoff of 0.58 (1.5-fold change), respectively. Proteins denoted by red and blue dots are significantly up- and downregulated, respectively, in Isoquercitrin_UV samples relative to VitaminC_UV samples. **(d)** Pathways downregulated in human fibroblasts upon UV exposure, in the absence of any treatment. **(e,f)** Pathways up- and downregulated in isoquercitriin-treated fibroblasts relative to vitamin-C-treated fibroblasts, respectively, in the absence of UV exposure. **(g,h)** Pathways upregulated in Isoquercitrtn_UV samples relative to Vehicle_UV (no treatment) and VitaminC_UV samples.

Flavonoids, naturally upregulated in stressed plants, can protect against ultraviolet UV radiation (3, 28), and IDPs have been recently shown to protect organisms against environmental stressors like desiccation by forming fibers (20, 29). Thus, we investigated whether the ability of flavonoids to form supramolecular structures contributes to this protection by assessing the ability of quercetin, quercitrin, and isoquercitrin to protect cultured human fibroblasts from exposure to UV radiation. Treatment with these flavonoids resulted in concentration-dependent protection against the loss of cell viability caused by UV radiation, aligning with our previous studies (Figure 4a). Notably, flavonoids with glycosidic groups demonstrated the most robust protective effects, supporting our MDS finding that showed enhanced assembly of multidimensional structures by flavonoids containing sugar moieties.

Many of the protective effects of flavonoids have been previously attributed to antioxidant effects (1). Antioxidant effects can result from multiple mechanisms (30), so we conducted a proteomics study comparing the effects of flavonoids and the well-known antioxidant Vitamin C, which is a free radical scavenger (31), to understand the underlying protective pathways. Our PCA plot (Figure 4b) shows that cells treated with either Vitamin C or the flavonoid isoquercitrin, align with the untreated control on the PC1 axis, distinct from UV-treated cells, which implies a protective effect. Additionally, a more focused PCA plot comparing only cells treated with Vitamin C or flavonoid shows clear delineation based on treatment type (Supplementary Figure 3a), indicating that the cell protective mechanisms activated by these two treatments are distinct. Indeed, differential expression analysis showed distinct protein expression patterns between cells pretreated with flavonoids or Vitamin C (Figure 4c) before being exposed to UV light as well as between untreated cells with and without UV exposure (Supplementary Figure 3b). UV radiation upregulated cell death/senescence/necroptosis pathways (Supplementary Figure 3c), and downregulated collagens and proteins associated with cell cycle/DNA synthesis/repair (Figure 4d; Supplementary Figure 3b) in the absence of flavonoid. Under control conditions without UV treatment, the flavonoid isoquercitrin upregulated proteins associated with DNA repair pathways and downregulated inflammatory pathways (Figures 4e,f; Supplementary Figure 3d), while Vitamin C showed opposite effects (Supplementary Figures 3e–g). When exposed to UV radiation, the presence of isoquercitrin induced upregulation of collagen and cell cycle/DNA repair proteins while downregulating proteins associated with autophagy, necroptosis, ferroptosis, lysosomal degradation, and other pathways (Figures 4g,h; Supplementary Figures 4h,i), whereas UV irradiated cells treated with Vitamin C primarily upregulated proteins associated with cell cycle/DNA repair-related pathways (Supplementary Figure 3k). Moreover, Vitamin C treatment resulted in lower expression of proteins involved in pathways associated with cellular response to reactive oxygen species, which can be generated by exposure to UV radiation. These included STK26, which is involved in the regulation of response to reactive oxygen species (GO:1901031, GO:0000302), response to hydrogen peroxide (GO:0042542), regulation of oxidative stress-induced cell death (GO:1903201), and SUMF1, which is involved in protein oxidation (GO:0018158) and oxidoreductase activity (GO:0016670) (32–35).

Notably, a comparison between isoquercitrin and Vitamin C in UV-irradiated cells revealed differential expression of various types of structural proteins (e.g., extracellular matrix, collagen, anchoring fibrils, etc.) and specifically, proteins involved in the supramolecular fiber organization pathway (GO:0097435) (32, 33) (Figure 4h). These results suggest that isoquercitrin may prevent the degradation of proteins found within regulatory pathways associated with higher-order structures, which is consistent with our models that predict isoquercitrin itself physically forms supramolecular structures and can interact physically with multiple proteins. These differences in protein expression patterns displayed by UV-irradiated cells treated with isoquercitrin versus Vitamin C raise the possibility that isoquercitrin enables cellular resilience to UV radiation by preventing the degradation of these multimolecular structural assemblies. Most importantly, our findings demonstrate that flavonoids and Vitamin C protect against UV radiation through different mechanisms and emphasize the role of flavonoids in supporting higher-order structural assemblies in a cellular context.

## Discussion

Flavonoids, widely consumed in plant-based diets, have long been associated with beneficial effects on inflammation, oxidative stress, and cellular resilience. Yet, the molecular mechanisms underlying these protective effects remain unknown. Here, we present data in support of a hypothesis that flavonoids may promote resilience by forming supramolecular assemblies that physically interact with protein structures (particularly in disordered regions) and influence enzyme function. These interactions appear to be influenced by flavonoid glycosylation, which modulates the dimensionality and compactness of their self-assembled structures.

Our simulations set the flavonoid concentration based on stoichiometry with the protein by molecular weight (134 mM and 81 mM for MARK4 and PTP1B, respectively). These concentrations were selected to ensure adequate stoichiometry while maintaining a simulation system size that is computationally pragmatic. Previous work (18, 19) showed how high and physiologically representative concentrations (2–8 mM) of ATP provide biological hydrotropic properties (19), which support soluble and active forms of intracellular and extracellular proteins. Our effective concentration in the biochemical and cellular assays was in the sub-100 μM range, which is still higher than dietary serum levels but may reflect locally elevated concentrations due to self-assembly, a hypothesis that remains to be validated in living tissues. A reason for the *in vitro* effect we observed at significantly lower concentrations may be the concentrating effect of self-assembly: as the molecules self-assemble around the protein, the effective local concentration within the vicinity of the protein is higher than that of the bulk solvent.

While flavonoids are often dismissed in drug discovery due to concerns about non-specific binding or aggregation flagged by PAINS filters (11, 36, 37), our findings suggest that their supramolecular assembly may represent a structured and biologically relevant mechanism of action (38–40). Rather than being artifacts, these assemblies could underlie functional modulation of protein dynamics and cellular stress responses. To minimize assay interference, we employed a label-free SAMDI-MS approach that is not susceptible to common artifacts such as redox cycling, autofluorescence, or absorbance interference (41, 42) often associated with polyphenolic compounds.

While our molecular dynamics simulations provide visual and structural insight into the behavior of flavonoid assemblies, they are exploratory in nature. Our goal here is to generate hypotheses, not to establish causality. While proximity-based effects are observed, further studies are needed to confirm functional consequences. Quantitative analyses such as RMSD, contact frequency, and free energy calculations were beyond the scope of this exploratory study but represent important future directions. We selected RGYR to assess overall shape changes and structural heterogeneity. RGYR provides a useful metric for future comparisons to experimental ion mobility (43–45) studies, which we plan to conduct. Ion mobility can reveal cross-sectional area and stoichiometry across a population of supramolecular ensembles. Quantitative characterization of binding modes, structural rearrangements, and stability—such as RMSD, contact analysis, or ensemble convergence—will require future analytical modeling using more advanced methods in addition to experimental validation. Our goal here is not to confirm molecular mechanisms but to generate hypotheses about potential supramolecular behaviors that can inform future studies.

Our studies are consistent with those of others who have recently observed the self-assembly of flavonoids and their capacity to form complex structures through non-covalent interactions (38). These studies have shown that due to the polyphenolic nature of flavonoids, they engage in hydrogen bonding, hydrophobic interactions, and van der Waals forces, which facilitate their aggregation. Specifically, it was demonstrated that the phenolic hydroxyl groups of flavonoids significantly influence their self-assembly with proteins, affecting the nature of these interactions (38). Past molecular dynamics simulations also have shown that flavonoids can form aggregates with polysaccharides, resulting in porous structures in aqueous solutions (39). Additionally, it has been shown that flavonoids can be absorbed into nano-colloids (40, 46) and coordination of flavonoids with metal ions can form complex materials influenced by environmental factors, such as pH and temperature (47). Crystal engineering of flavonoids has also been shown to induce the formation of extended hydrogen-bonded networks (48). Our findings extend this field by demonstrating that flavonoid assemblies can take on highly ordered forms and play a role in the expression of enzyme structural protein assemblies under environmental stresses that provide increased cell resilience, preventing damage in response to UV radiation exposure. A full list of enriched pathways is available in the Supplementary data, and further analysis across tissues will be important in future work.

This study proposes a novel hypothesis: dietary flavonoids form supramolecular structures that influence protein conformation and enhance stress resilience. Our initial data support this idea and open a new direction in nutritional biochemistry. Importantly, our findings support the notion that these compounds act through mechanisms distinct from classical antioxidant activity—namely, through structural modulation and potential stabilization of disordered or stress-sensitive protein assemblies. These preliminary findings point toward a broader framework in which dietary flavonoid diversity promotes resilience through heterogeneous, multi-target biochemical modulation. Future work will involve more rigorous structural modeling, experimental validation, and testing across diverse nutritional contexts and cell types.

## Materials and methods

### Enzyme assays

Enzyme screens were performed by SAMDI Tech (contract research organization); enzyme assays were performed in a 20 μL volume in 384-well low-volume polypropylene microtiter plates (Greiner Bio-One) at room temperature. The enzymes [Arginase 1 (ARG1), Lysine demethylase 4C (KDM4C), MAP/microtubule affinity-regulating kinase 4 (MARK4), Histone-Lysine N-methyltransferase (NSD2), Tyrosine-protein phosphatase non-receptor type 1 (PTP1B), and Sirtuin-3 (SIRT3)] were incubated with compounds for 30 min prior to initiation of the reaction by the addition of substrate and cofactors (if required). Reactions were quenched by the addition of 0.5% formic acid (final) with subsequent neutralization using 1% sodium bicarbonate (final). For SAMDI MS analysis, 2 μL of each reaction was transferred using a 384-channel automated liquid handler to SAMDI biochip arrays functionalized with a neutravidin-presenting self-assembled monolayer. The preparation of SAMDI biochip arrays has been previously described (41, 42, 49). The SAMDI arrays were incubated for 1 h in a humidified chamber to allow specific immobilization of the biotinylated substrates and products; they were then purified by washing with ultrafiltered water (50 μL/spot) and dried with compressed air. A matrix comprising α-Cyano-4-hydroxycinnamic acid in 80% acetonitrile: 20% aqueous ammonium citrate (10 mg/mL final) was applied in an automated format by dispensing 50 nL to each spot in the array. SAMDI MS was performed using reflector positive mode on an AB Sciex TOF-TOF 5800 System (AB Sciex, Framingham, MA) with 400 shots/spot analyzed in a random raster sampling. For data analysis, the area under the curves (AUCs) for the product peak and substrate peak were calculated using the TOF/TOF Series Explorer (AB Sciex), and the amount of product formed was calculated using the equation AUC _product_ / (AUC _product_ + AUC _Substrate_). Negative controls were pre-quenched with 0.5% formic acid (final). Lysozyme assay was performed using the manufacturer’s instructions (Biovision Lysozyme inhibitor screening kit K237). Flavonoids were acquired from Sigma-Aldrich and Seleckchem.

### Enzyme assay analysis

*IC50 values of enzyme inhibition.* Inhibition of the enzymes was measured at 10 concentration values in the range of 5 nM to 100 μM for each compound and enzyme, with two technical replicates; the percent inhibition values were determined by SAMDI software. Sigmoidal curves were fitted to the percent inhibition *vs* log_10_ (concentration) series, and IC50 values were determined at the crossing point of 50 % inhibition. Manual verification of the fits and the IC50 values was performed. The resulting IC50 values fell into the following categories: (a) within the assayed range of 5nM to 100μM (26 % of fits), (b) IC50 could be extrapolated, IC50 > 100μM (12 % of fits), or (c) fitting was not possible because all values were at baseline (62 % of fits). *Compound efficacy ranking*. First, for each IC50 value per compound and enzyme, a Z-score was calculated based on the log_10_ (*IC50*) value and that of the compounds that produced a successful readout for enzyme i , according to Equation 1:


(1) $$z_{i}=\frac{\log_{10}(\mathrm{IC}50_{i})-\bar{X_{i}}}{s_{i}}$$

Where $\bar{X_{i}}$ and $s_{i}$ are the average of all successfully determined log_10_ (*IC50*) values for enzyme i . Any compound that did not produce a readout was assigned z=2 , this imputation allows ranking of compounds with very high IC50 values. Then, for each compound, a weighted mean of the z-scores ( $\bar{z}$ ) was calculated according to Equation 2:


(2) $$\bar{z}=\frac{\sum_{i=1}^{i=7}w_{i}z_{i}}{7}$$

where the weights were calculated according to Equation 3:


(3) $$w_{i}=\frac{\sqrt{N_{i}}}{\sqrt{\sum_{i=1}^{i=7}N_{i}}}$$

Where $N_{i}$ is the number of successful readouts for enzyme i . Finally, for those compounds that had multiple repeated readouts, the average of the $\bar{z}$ values was used. Compounds were ranked based on the $\bar{z}$ values: low $\bar{z}$ values mean high efficacy of inhibition, and high values mean low efficacy. Fitting was performed using R (v3.6.3) and the gslnls package (v1.1.1), and figures were rendered using the ggplot2 package (v3.4.0).

### Molecular dynamics simulation

For the MARK4and PTP1B simulations, initial protein structures were obtained from UniProt AlphaFoldDB for MARK4 (AF-Q96L34-F1) and PTP1B (AF-P18031-F1). Flavonoid structures were acquired from PubChem (quercetin CID-5280343, isoquercitrin CID-5280804, and quercitrin CID-5280459). Initial state input files for simulation were generated using Packmol (50), where a 150 Å box was used to randomly distribute 273 flavonoid molecules with MARK4 and 165 for PTP1B. Water was added with a balanced charge of Na+ and Cl- ions, plus 250 ions of each randomly placed using AmberTools (51). AmberTools tleap generated the system with the amber forcefields of tip3, ff14SB, and gaff2. Simulations were carried out with OpenMM (52). Periodic boundary conditions with a Langevin integrator were used with a PME non-bonded method, a non-bonded cutoff of 1 nm, Ewald error tolerance of 0.0005, and non-rigid water. A constant temperature of 310 K and pressure of 1 atm were maintained with a Montecarlo barostat at an interval of 25. MD simulations of proteins with flavonoids were energy minimized and equilibrated for 1 ns before being simulated for >400 ns. These simulations were run in duplicate.

The simulation inputs for lysozyme and quercetin were generated the same way, with a 150 Å box, two lysozyme molecules, 128 quercetin, Na+, and Cl- ions to balance charge, with an additional 74 of each ion. The starting coordinates were from PDB: 193l. The explicit solvent system with periodic boundary conditions was generated and simulated as above for 400 ns. Visualization of the force distribution was performed using Houdini software, and the force value for each atom was taken from the final state of the simulation. The length of the force vector was then normalized and used to assign a color. These visualizations were to provide qualitative insight and were not intended to make quantitative claims about structural stability. Simulations with ATP, ADP, and Flavonoid consisted of a 40 Å box with 10 flavonoid molecules, 5 ADP, 5 ATP, and a balanced charge with 5 additional Cl- and Na+ ions, generated as above and simulated for 100 ns in triplicate for quercetin, quercitrin, and isoquercitrin.

RGYR analysis was performed using the Python package mdtraj (53). Visualizations of protein structures were produced using Houdini and the Python packages prody (54), mdtraj, and biopython (55). Input files for weighted ensemble simulations were generated again with Packmol. A 40 Å water box, a balanced charge of Na+ and Cl– ions, plus 5 ions of each randomly placed before being carried out using WEPY (26) with 50 walkers, 1000 cycles, 10 ps steps, the REVOResampler, and an RGYR distance function. Final walker lineages and probability distribution were calculated based on the Lotz et al. (26). pipeline and an RGYR distance function (26). Pre-simulation energy minimization was performed. The same forcefields and periodic boundary parameters were used as above, with a constant temperature of 300 K. REVOresampler merge distance of 0.25, char_dist of 0.1, pmax 0.5, pmin 1e-12, and a dist_exp of 4. Levene’s test for statistical analysis of variance was performed using the Python library Scipy on the RGYR values between 200 and 400 ns. The duplicate RGYR values were combined, and each pair of simulations for each flavonoid was compared with the control simulations with no flavonoid to provide a *p*-value for the significance of variation. *p*-values of less than 0.005 were achieved in all cases. Violin plots were generated with the Python packages matplotlib and seaborn with the same values from 200 to 400 ns.

### Cell culture and ultraviolet radiation

Human fibroblasts were obtained from Cell Applications Inc (Cat. # 106-05a) and were maintained in Dulbecco’s Modified Eagle medium (DMEM, Corning, Cat. #10-013-CV) with 10% fetal bovine serum (Gibco, Cat. #10082147) and 1% penicillin/streptomycin (Gibco, Cat. # 15070-063) in a humidified incubator with 5% CO_2_ at 37 °C. For ultraviolet radiation, fibroblasts were seeded in a 96-well plate at a density of 25,000 cells/well and maintained at 37 °C for 24 h. Cells were washed once with Hanks’ balanced salt solution (HBSS, Gibco, Cat. #14025-092), then treated for 1 h with different concentrations of compounds prepared in HBSS. Vitamin C (Sigma, Cat. # A5960) served as a positive control. Treated cells were exposed to ultraviolet light (UVP cross-linker, CL1000, 254 nm UV, 8 watts) at a total dose of 20 mJ/cm^2^. Following irradiation, the HBSS was replaced with culture medium containing the same concentration of compounds as before irradiation, and cells were again maintained at 37 °C. Cell viability was measured at 24 h post-irradiation using CellTiter-Glo according to the manufacturer’s instructions (Promega, Cat.# G7571). Results were expressed as relative cell viability (%) with respect to control cells (cells without ultraviolet irradiation or compound treatment).

### Proteomics sample preparation

Frozen cell pellets were dissolved for 15 min in 6M Guanidinium Chloride at room temperature to lyse cells. Then, each solution was run on a 10 kDa filter (Pall, TX) and washed with 50 μL of TEAB buffer at 3000 rpm. FASP Digestion (56): A stock solution of Trypsin Platinum, Mass Spec Grade (Promega) at 100 μg/mL in 50 mM TEAB, and a specific volume of stock trypsin was added to each sample vial for each set of brain cells using a 1:50 ratio (Trypsin:Protein). Samples were incubated for 2 h at 50 °C and shaken/mixed at 350 rpm on an Eppendorf ThermoMixer C. TMT Labeling: Samples, now peptides, were labeled using 4 μL of TMTpro Mass Tag Labels (ThermoScientific). After labeling, samples were then shaken/mixed for 45 min to ensure labels were covalently bonded to peptides. The labeling reaction was quenched for 10 min using 1 μL of 5% hydroxyalamine. After quenching, samples were pooled into one 2 mL Eppendorf tube and then dried down using an Eppendorf Vacufuge Plus. Desalting Samples: Dried samples were resuspended in 300 μL of 0.1% TFA in ultrapure HPLC-grade water and vortexed to ensure full solubility. Samples were then desalted using Pierce^TM^ Peptide Desalting Spin Columns (ThermoScientific). Final eluates (desalted samples) contained 600 μL of 50% HPLC-grade Acetonitrile and 50% ultrapure HPLC-grade water. Desalted samples were then transferred to HPLC vials and were again dried down. Dried and desalted samples were resuspended in 6 μL of 0.1% Formic Acid in ultrapure HPLC-grade water and were then injected for LC-MS/MS analysis.

### Mass spectrometry analysis

After separation, each fraction was submitted for a single LC-MS/MS experiment performed on an HFX Orbitrap (Thermo Scientific) equipped with an Exigent (SCIEX, MA) nanoHPLC pump. Peptides were separated onto a 150 μm x 8 cm PepSep C18 analytical column (Bruker, MA) by applying a gradient from 5 to 25% ACN in 0.1% formic acid over 90 min at 300 nL min−1. Electrospray ionization was enabled by applying a voltage of 2 kV using a PepSep electrode junction at the end of the analytical column and sprayed from a stainless steel PepSep emitter SS 30 μm LJ (Odense, Denmark). The HF Orbitrap was operated in data-dependent mode for the mass spectrometry methods. The mass spectrometry survey scan was performed in the Orbitrap in the range of 450 –900 m/z at a resolution of 1.2 × 10^5^, followed by selection of the ten most intense ions. The (TOP10) ions were then subjected to an HCD MS2 event in the Orbitrap part of the instrument. The fragment ion isolation width was set to 0.8 m/z, AGC was set to 50,000, the maximum ion time was 150 ms, normalized collision energy was set to 34V, and an activation time of 1 ms was set for each HCD MS2 scan.

### Mass spectrometry data analysis

Raw data were submitted for analysis in Proteome Discoverer 3.0.1.23 (Thermo Scientific) software with Chimerys. Assignment of MS/MS spectra was performed using the Sequest HT algorithm and Chimerys (MSAID, Germany) by searching the data against a protein sequence database including all entries from the Mouse Uniprot database (SwissProt 19,768 2019) and other known contaminants such as human keratins and common lab contaminants. Sequest HT searches were performed using a 20 ppm precursor ion tolerance and requirement of each peptide N-/C termini to adhere with Trypsin protease specificity while allowing up to two missed cleavages. 18-plex TMT tags on peptide N termini and lysine residues (+304.207146 Da) were set as static modifications, and Carbamidomethyl on cysteine amino acids (+57.021464 Da), while methionine oxidation (+15.99492 Da) was set as a variable modification. An MS2 spectra assignment false discovery rate (FDR) of 1% on the protein level was achieved by applying the target-decoy database search. Filtering was performed using a Percolator (64-bit version) (57). For quantification, a 0.02 m/z window centered on the theoretical m/z value of each of the six reporter ions, and the intensity of the signal closest to the theoretical m/z value was recorded. Reporter ion intensities were exported to the result file of the Proteome Discoverer 3.0 search engine as Excel tables. The total signal intensity across all peptides quantified was summed for each TMT channel, and all intensity values were adjusted to account for potentially uneven TMT labeling and/or sample handling variance for each labeled channel.

### Proteomics analysis

Protein level data from each TMT channel was analyzed in R. The dataset was first preprocessed by removing contaminant proteins and those with unavailable abundance data. Next, the dataset was normalized using the variance stabilizing normalization method (vsn) with the R MSnbase package. Differential expression (DE) analysis utilized the R limma package with volcano plots for visualization (pval ≤ 0.05, log2fc ≥ |0.58| for significance). Only the proteins identified as significantly differentially expressed were selected for further pathway analysis, which was performed with the R Enrichr package, identifying significantly altered pathways (pval ≤ 0.05) for up- and downregulated proteins in each DE comparison. The proteomic data set is available on the massive data repository (MSV000095599).

## Data Availability

Proteomics data has been deposited in MassIVE with the dataset ID MSV000095599 and can be accessed at https://massive.ucsd.edu/ProteoSAFe/private-dataset.jsp?task=2c6f205104f74fb799eea121bfc415b3. All other data generated or analyzed during this study are included in the published article.

## References

[1] Pietta PG. Flavonoids as antioxidants. J Nat Prod. (2000) 63:1035–42. doi: 10.1021/np9904509, PMID: 10924197

[2] Panche AN Diwan AD Chandra SR. Flavonoids: an overview. J Nutr Sci. (2016) 5:e47. doi: 10.1017/jns.2016.41, PMID: 28620474 PMC5465813

[3] Casagrande R Georgetti SR Verri WA Jr Dorta DJ dos Santos AC Fonseca MJV. Protective effect of topical formulations containing quercetin against UVB-induced oxidative stress in hairless mice. J Photochem Photobiol B. (2006) 84:21–7. doi: 10.1016/j.jphotobiol.2006.01.006, PMID: 16495072

[4] Moon J-K Shibamoto T. Antioxidant assays for plant and food components. J Agric Food Chem. (2009) 57:1655–66. doi: 10.1021/jf803537k, PMID: 19182948

[5] Parmenter BH Thompson AS Bondonno NP Jennings A Murray K Perez-Cornago A. High diversity of dietary flavonoid intake is associated with a lower risk of all-cause mortality and major chronic diseases. Nat Food. (2025) 6:668–80. doi: 10.1038/s43016-025-01176-1, PMID: 40456886 PMC12283405

[6] Hong Y Wang Z Barrow CJ Dunshea FR Suleria HAR. High-throughput screening and characterization of phenolic compounds in stone fruits waste by LC-ESI-QTOF-MS/MS and their potential antioxidant activities. Antioxidants (Basel). (2021) 10:234. doi: 10.3390/antiox10020234, PMID: 33557299 PMC7914583

[7] Li Y Yao J Han C Yang J Chaudhry M Wang S. Quercetin, inflammation and immunity. Nutrients. (2016) 8:167. doi: 10.3390/nu8030167, PMID: 26999194 PMC4808895

[8] Lin Y Shi R Wang X Shen H-M. Luteolin, a flavonoid with potential for cancer prevention and therapy. Curr Cancer Drug Targets. (2008) 8:634–46. doi: 10.2174/156800908786241050, PMID: 18991571 PMC2615542

[9] Moghaddam E. Baicalin, a metabolite of baicalein with antiviral activity against dengue virus. Sci Rep. (2014) 4:1–8. doi: 10.1038/srep05452 PMC4071309 24965553

[10] Hu Z Guan Y Hu W Xu Z Ishfaq M. An overview of pharmacological activities of baicalin and its aglycone baicalein: new insights into molecular mechanisms and signaling pathways. Iran J Basic Med Sci. (2022) 25:14–26. doi: 10.22038/IJBMS.2022.60380.13381, PMID: 35656442 PMC9118284

[11] Baell JB Nissink JWM. Seven year itch: Pan-assay interference compounds (PAINS) in 2017-utility and limitations. ACS Chem Biol. (2018) 13:36–44. doi: 10.1021/acschembio.7b00903, PMID: 29202222 PMC5778390

[12] Baell JB Holloway GA. New substructure filters for removal of pan assay interference compounds (PAINS) from screening libraries and for their exclusion in bioassays. J Med Chem. (2010) 53:2719–40. doi: 10.1021/jm901137j, PMID: 20131845

[13] Watkins BA Mitchell AE Shin AC Dehghani F Shen C-L. Dietary flavonoid actions on senescence, aging, and applications for health. J Nutr Biochem. (2025) 139:109862. doi: 10.1016/j.jnutbio.2025.109862, PMID: 39929283

[14] Jumper J Evans R Pritzel A Green T Figurnov M Ronneberger O. Highly accurate protein structure prediction with AlphaFold. Nature. (2021) 596:583–9. doi: 10.1038/s41586-021-03819-2, PMID: 34265844 PMC8371605

[15] Jiang Y Li X Morrow BR Pothukuchy A Gollihar J Novak R. Single-molecule mechanistic study of enzyme hysteresis. ACS Cent Sci. (2019) 5:1691–8. doi: 10.1021/acscentsci.9b00718, PMID: 31660437 PMC6813718

[16] Gilboa T Ogata AF Reilly CB Walt DR. Single-molecule studies reveal method for tuning the heterogeneous activity of alkaline phosphatase. Biophys J. (2022) 121:2027–34. doi: 10.1016/j.bpj.2022.05.005, PMID: 35527401 PMC9247479

[17] Ehrl BN Liebherr RB Gorris HH. Single molecule kinetics of horseradish peroxidase exposed in large arrays of femtoliter-sized fused silica chambers. Analyst. (2013) 138:4260–5. doi: 10.1039/c3an00809f, PMID: 23752650

[18] Takaine M Imamura H Yoshida S. High and stable ATP levels prevent aberrant intracellular protein aggregation in yeast. eLife. (2022) 11:e67659. doi: 10.7554/eLife.67659, PMID: 35438635 PMC9018071

[19] Patel A Malinovska L Saha S Wang J Alberti S Krishnan Y. ATP as a biological hydrotrope. Science. (2017) 356:753–6. doi: 10.1126/science.aaf6846, PMID: 28522535

[20] Malki A Teulon JM Camacho-Zarco AR Chen SWW Adamski W Maurin D. Intrinsically disordered tardigrade proteins self-assemble into fibrous gels in response to environmental stress. Angew Chem Int Ed Eng. (2022) 61:e202109961. doi: 10.1002/anie.202109961, PMID: 34750927 PMC9299615

[21] Wang N Tytell JD Ingber DE. Mechanotransduction at a distance: mechanically coupling the extracellular matrix with the nucleus. Nat Rev Mol Cell Biol. (2009) 10:75–82. doi: 10.1038/nrm2594, PMID: 19197334

[22] Ingber DE. Cellular mechanotransduction: putting all the pieces together again. FASEB J. (2006) 20:811–27. doi: 10.1096/fj.05-5424rev, PMID: 16675838

[23] Jaalouk DE Lammerding J. Mechanotransduction gone awry. Nat Rev Mol Cell Biol. (2009) 10:63–73. doi: 10.1038/nrm2597, PMID: 19197333 PMC2668954

[24] Wang N Butler JP Ingber DE. Mechanotransduction across the cell surface and through the cytoskeleton. Science. (1993) 260:1124–7. 7684161 10.1126/science.7684161

[25] Watson JD Crick FH. The structure of DNA. Cold Spring Harb Symp Quant Biol. (1953) 18:123–31. 13168976 10.1101/sqb.1953.018.01.020

[26] Lotz SD Dickson A. Wepy: a flexible software framework for simulating rare events with weighted ensemble resampling. ACS Omega. (2020) 5:31608–23. doi: 10.1021/acsomega.0c03892, PMID: 33344813 PMC7745226

[27] Jin MY Zhen Q Xiao D Tao G Xing X Yu P. Engineered non-covalent π interactions as key elements for chiral recognition. Nat Commun. (2022) 13:3276. doi: 10.1038/s41467-022-31026-8, PMID: 35672365 PMC9174283

[28] Nascimento LBDS Leal-Costa MV Menezes EA Lopes VR Muzitano MF Costa SS. Ultraviolet-B radiation effects on phenolic profile and flavonoid content of *Kalanchoe pinnata*. J Photochem Photobiol B. (2015) 148:73–81. doi: 10.1016/j.jphotobiol.2015.03.011, PMID: 25900552

[29] Urosev I Lopez Morales J Nash MA. Phase separation of intrinsically disordered protein polymers mechanically stiffens fibrin clots. Adv Funct Mater. (2020) 30:2005245. doi: 10.1002/adfm.202005245

[30] Lü J-M Lin PH Yao Q Chen C. Chemical and molecular mechanisms of antioxidants: experimental approaches and model systems. J Cell Mol Med. (2010) 14:840–60. doi: 10.1111/j.1582-4934.2009.00897.x, PMID: 19754673 PMC2927345

[31] Njus D Kelley PM Tu Y-J Schlegel HB. Ascorbic acid: the chemistry underlying its antioxidant properties. Free Radic Biol Med. (2020) 159:37–43. doi: 10.1016/j.freeradbiomed.2020.07.013, PMID: 32738399

[32] Ashburner M Ball CA Blake JA Botstein D Butler H Cherry JM. Gene ontology: tool for the unification of biology. Nat Genet. (2000) 25:25–9. doi: 10.1038/75556, PMID: 10802651 PMC3037419

[33] Gene Ontology Consortium. The gene ontology knowledgebase in 2023. Genetics. (2023) 224:iyad031. doi: 10.1093/genetics/iyad031 PMC10158837 36866529

[34] Liu Q Yu M Zhang T. Construction of oxidative stress-related genes risk model predicts the prognosis of uterine Corpus endometrial Cancer patients. Cancer. (2022) 14:5572. doi: 10.3390/cancers14225572, PMID: 36428665 PMC9688652

[35] Pierzynowska K Gaffke L Cyske Z Węgrzyn G Buttari B Profumo E. Oxidative stress in Mucopolysaccharidoses: pharmacological implications. Molecules. (2021) 26:5616. doi: 10.3390/molecules26185616, PMID: 34577086 PMC8468662

[36] McGovern SL Caselli E Grigorieff N Shoichet BK. A common mechanism underlying promiscuous inhibitors from virtual and high-throughput screening. J Med Chem. (2002) 45:1712–22. doi: 10.1021/jm010533y, PMID: 11931626

[37] Dahlin JL Nelson KM Strasser JM Barsyte-Lovejoy D Szewczyk MM Organ S. Assay interference and off-target liabilities of reported histone acetyltransferase inhibitors. Nat Commun. (2017) 8:1527. doi: 10.1038/s41467-017-01657-3, PMID: 29142305 PMC5688144

[38] Wang X-J. Regulation mechanism of phenolic hydroxyl number on self-assembly and interaction between edible dock protein and hydrophobic flavonoids. J Agric Food Chem. (2023) 71:18510–23. doi: 10.1021/acs.jafc.3c05713, PMID: 37971491

[39] Yang B Wu X Zeng J Song J Qi T Yang Y. A multi-component nano-co-delivery system utilizing Astragalus polysaccharides as carriers for improving biopharmaceutical properties of Astragalus flavonoids. Int J Nanomedicine. (2023) 18:6705–24. doi: 10.2147/IJN.S434196, PMID: 38026532 PMC10656867

[40] Mandial D Khullar P Kumar H Ahluwalia GK Bakshi MS. Naringin–Chalcone bioflavonoid-protected Nanocolloids: mode of flavonoid adsorption, a determinant for protein extraction. ACS Omega. (2018) 3:15606–14. doi: 10.1021/acsomega.8b01776, PMID: 31458217 PMC6643453

[41] Scholle MD Gurard-Levin ZA. Development of a novel label-free and high-throughput arginase-1 assay using self-assembled monolayer desorption ionization mass spectrometry. SLAS Discov. (2021) 26:775–82. doi: 10.1177/24725552211000677, PMID: 33754845

[42] Gurard-Levin ZA Scholle MD Eisenberg AH Mrksich M. High-throughput screening of small molecule libraries using SAMDI mass spectrometry. ACS Comb Sci. (2011) 13:347–50. doi: 10.1021/co2000373, PMID: 21639106 PMC3132997

[43] Du C. Combining surface-induced dissociation and charge detection mass spectrometry to reveal the native topology of heterogeneous protein complexes. Anal Chem. (2023) 95:13889–96. doi: 10.1021/acs.analchem.3c02185, PMID: 37672632 PMC10874503

[44] Kalenius E Groessl M Rissanen K. Ion mobility–mass spectrometry of supramolecular complexes and assemblies. Nat Rev Chem. (2018) 3:4–14. doi: 10.1038/s41570-018-0062-2

[45] Williams OHL. Automated structural activity screening of β-diketonate assemblies with high-throughput ion mobility-mass spectrometry. Angew Chem Weinheim Bergstr Ger. (2024) 136:e202313892. doi: 10.1002/anie.202313892 38012094

[46] Cui L Sun E Zhang ZH Tan XB Wei YJ Jin X. Enhancement of Epimedium fried with suet oil based on in vivo formation of self-assembled flavonoid compound Nanomicelles. Molecules. (2012) 17:12984–96. doi: 10.3390/molecules171112984, PMID: 23117437 PMC6268372

[47] Walencik PK. Metal–flavonoid interactions—from simple complexes to advanced systems. Molecules. (2024) 29:2573. doi: 10.3390/molecules29112573, PMID: 38893449 PMC11173564

[48] Timmons DJ Pacheco MR Fricke KA Slebodnick C. Assembling extended structures with flavonoids. Cryst Growth Des. (2008) 8:2765–9. doi: 10.1021/cg7009572

[49] Patel K Sherrill J Mrksich M Scholle MD. Discovery of SIRT3 inhibitors using SAMDI mass spectrometry. J Biomol Screen. (2015) 20:842–8. doi: 10.1177/1087057115588512, PMID: 26024947

[50] Martínez L Andrade R Birgin EG Martínez JM. PACKMOL: a package for building initial configurations for molecular dynamics simulations. J Comput Chem. (2009) 30:2157–64. doi: 10.1002/jcc.21224, PMID: 19229944

[51] Salomon-Ferrer R Case DA Walker RC. An overview of the Amber biomolecular simulation package. Wiley Interdiscip Rev Comput Mol Sci. (2013) 3:198–210. doi: 10.1002/wcms.1121

[52] Eastman P Swails J Chodera JD McGibbon RT Zhao Y Beauchamp KA. OpenMM 7: rapid development of high performance algorithms for molecular dynamics. PLoS Comput Biol. (2017) 13:e1005659. doi: 10.1371/journal.pcbi.1005659, PMID: 28746339 PMC5549999

[53] McGibbon RT Beauchamp KA Harrigan MP Klein C Swails JM Hernández CX. MDTraj: a modern open library for the analysis of molecular dynamics trajectories. Biophys J. (2015) 109:1528–32. doi: 10.1016/j.bpj.2015.08.015, PMID: 26488642 PMC4623899

[54] Bakan A Dutta A Mao W Liu Y Chennubhotla C Lezon TR. Evol and ProDy for bridging protein sequence evolution and structural dynamics. Bioinformatics. (2014) 30:2681–3. doi: 10.1093/bioinformatics/btu336, PMID: 24849577 PMC4155247

[55] Cock PJA Antao T Chang JT Chapman BA Cox CJ Dalke A. Biopython: freely available Python tools for computational molecular biology and bioinformatics. Bioinformatics. (2009) 25:1422–3. doi: 10.1093/bioinformatics/btp163, PMID: 19304878 PMC2682512

[56] Wiśniewski JR Zougman A Nagaraj N Mann M. Universal sample preparation method for proteome analysis. Nat Methods. (2009) 6:359–62. doi: 10.1038/nmeth.1322, PMID: 19377485

[57] Käll L Storey JD Stafford W. Nonparametric estimation of posterior error probabilities associated with peptides identified by tandem mass spectrometry. Bioinformatics. (2008) 24:i42–8. doi: 10.1093/bioinformatics/btn294, PMID: 18689838 PMC2732210

