# Supplementary material for: Dietary flavonoids form supramolecular assemblies, alter biochemistry, and enhance cell resilience
Source: Front Nutr. 2025 Sep 2;12:1649867. doi: 10.3389/fnut.2025.1649867 (PMC12439166; doi:10.3389/fnut.2025.1649867)
Supplement: Supplementary file 2 [file Data_Sheet_1.pdf]

Supporting Information for:

Dietary flavonoids form supramolecular assemblies, alter biochemistry, and enhance cell resilience

Charles B. Reilly[1], Sylvie G. Bernier[1], Sanjid Shahriar[1], Viktor Horvath[1], Michael Lewandowski[1], Emilia Javorsky[1], Bogdan Budnik[1], and Donald E. Ingber[1-3]*.

[1] Wyss Institute for Biologically Inspired Engineering at Harvard University, Boston, MA 02215, USA.
[2]Harvard John A. Paulson School of Engineering and Applied Sciences, Harvard University, Cambridge, MA 02138, USA.
[3]Vascular Biology Program and Department of Surgery, Harvard Medical School and Boston Children's Hospital, Boston, MA 02115, USA.
*Correspondence to: don.ingber@wyss.harvard.edu

name	Uniprot ID	Enzyme	structure	description
**MARK4**	Q96L34	Microtubule affinity regulating kinase 4	monomeric	serine/threonine phosphorylation
**KDM4C**	Q9H3R0	Lysine demethylase 4C	monomeric	demethylation of histones
**Lysozyme**	P00698	Lysozyme	monomeric	Breaks glycosidic bonds
**NSD2**	O96028	Histone-Lysine N-methyltransferase	monomeric	Epigenetic control of gene expression
**PTP1B**	P18031	Protein tyrosine phosphatase	monomeric	Negative regulator of insulin signaling pathway.
**ARG1**	P05089	Arginase I	trimeric	Converts arginine to ornithine and urea
**SIRT3**	Q9NTG7	Siturin-3	dimeric	Mitochondrial NAD-dependent deacetylase

Extended Data Table. 1: Enzymes used for biochemical screens.

Summary table of compound ranking and enzymatic activity assays

Name	Structure	Rank	ARG1	KDM4C	Lysozyme	MARK4	NSD2	PTP1B	SIRT3
quercetin	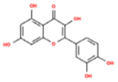	12.14							
isoquercitrin	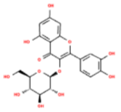	49.86							
orientin	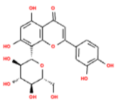	37.43							
mangiferin	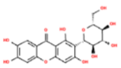	43.71							
gossypin	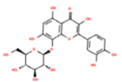	46.57							
tiliroside	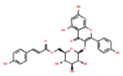	53.14							

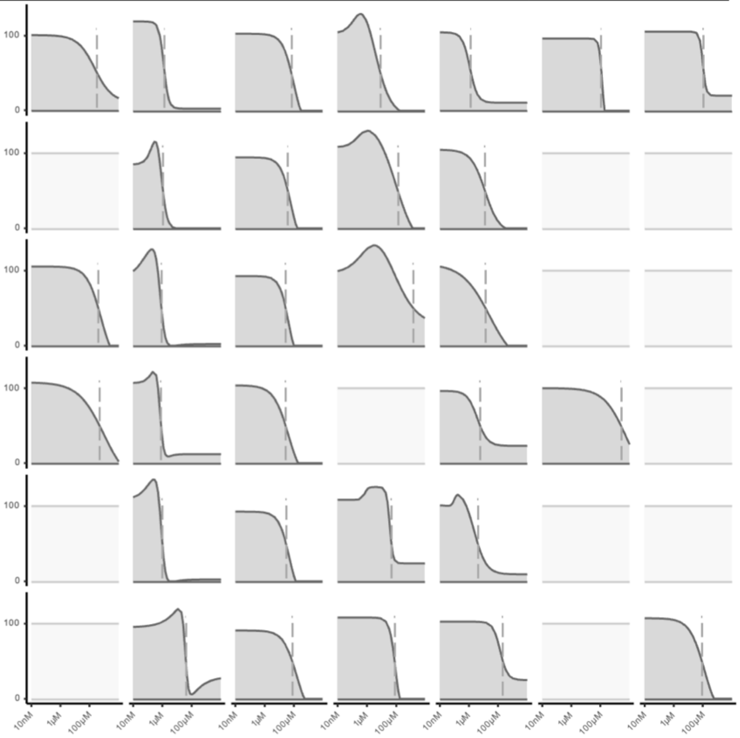

Extended Data Table. 2a.

Summary table of compound ranking and enzymatic activity assays

Name	Structure	Rank
leucoside	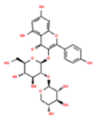	54.86
myricitrin	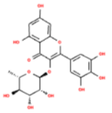	58.86
rutin	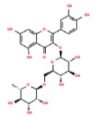	59.14
guaijaverin	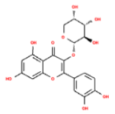	59.86
rhamnetin 3-sophoroside	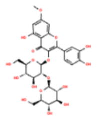	61.43
avicularin	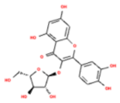	63.43

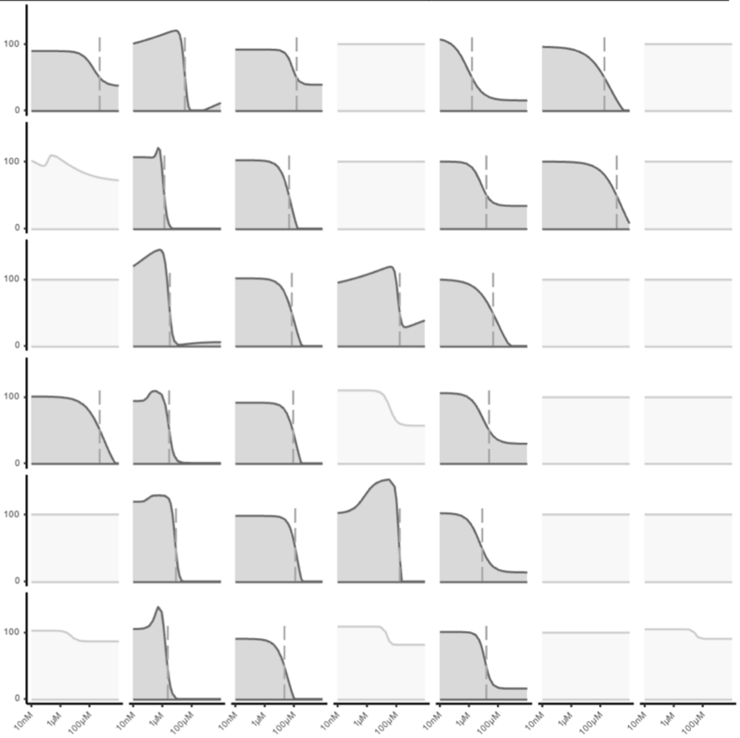

Extended Data Table. 2b.

Summary table of compound ranking and enzymatic activity assays

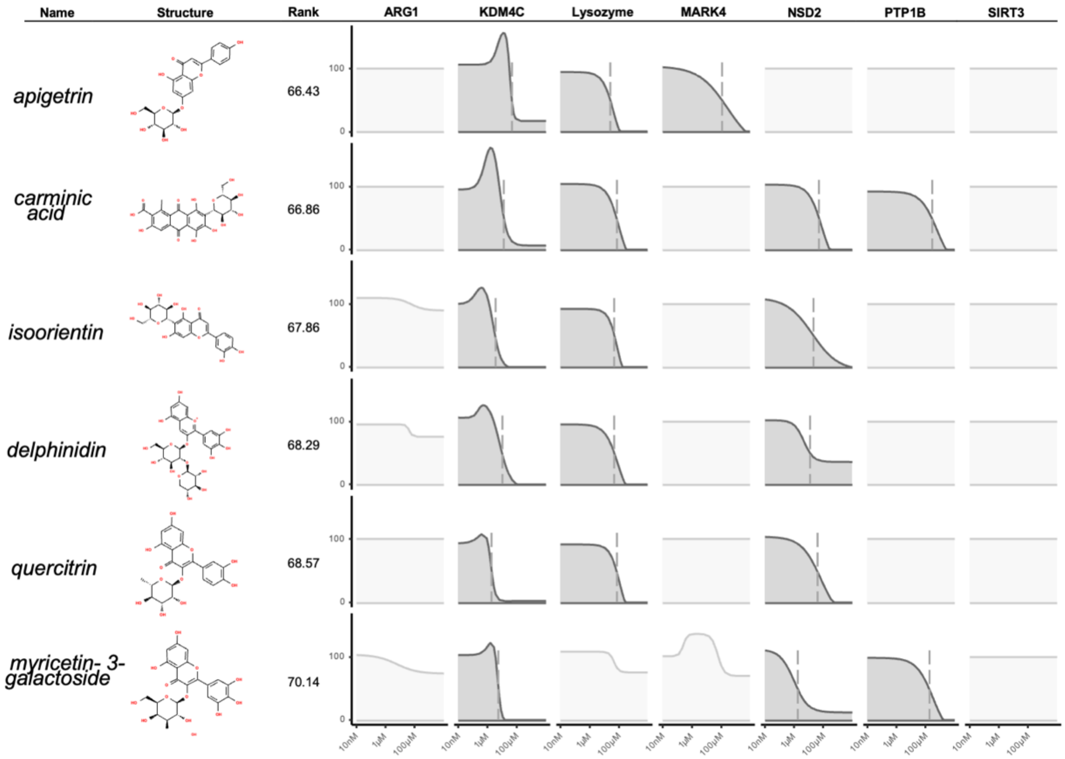

Extended Data Table. 2c.

Summary table of compound ranking and enzymatic activity assays

Name	Structure	Rank
baicalin	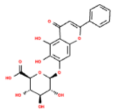	70.57
miquelianin	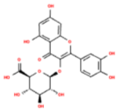	70.71
fluorescein mono-b-d-galactopyranoside	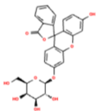	71.57
isorhamnetin-3-O-beta-D-glucotrioside	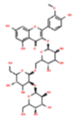	71.71
Quercetin 3-gentionbioside	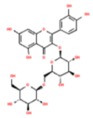	71.86
hyperoside	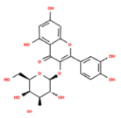	73.71

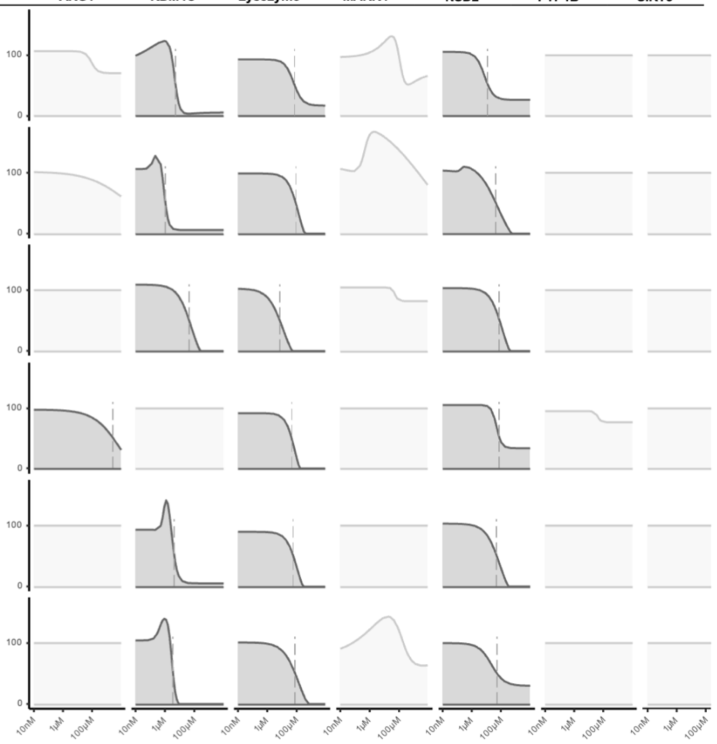

Extended Data Table. 2d.

Summary table of compound ranking and enzymatic activity assays

Name	Structure	Rank
Quercetin 3,4'-diglucoside	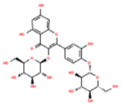	73.71
spiraeoside	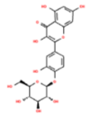	76.43
quercetin 3-o-malonylglucoside	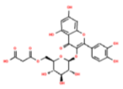	78.29
haploside C	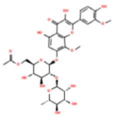	79
mearnsitrin	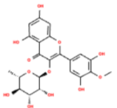	79
Peonidin-3-O-rutinoside chloride	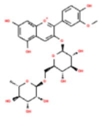	79.57

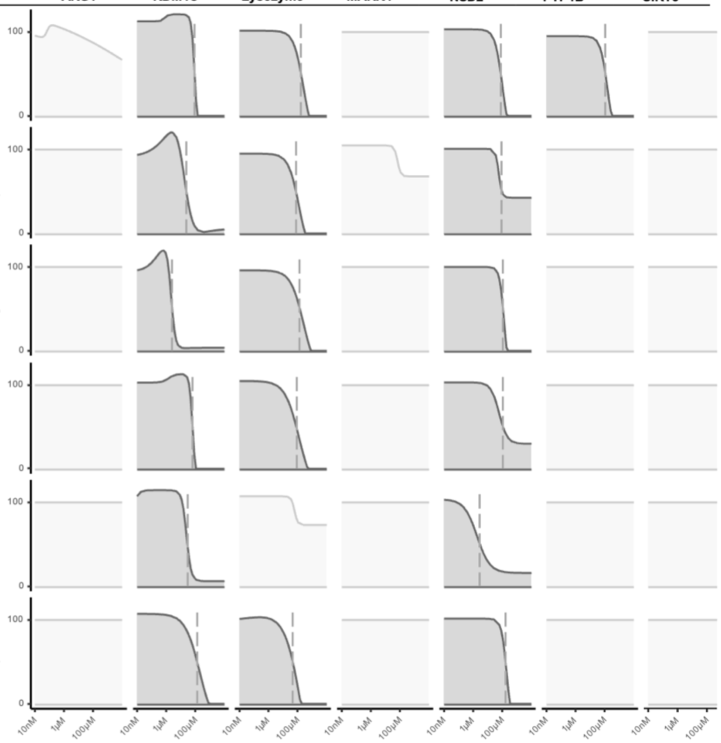

Extended Data Table. 2e.

Summary table of compound ranking and enzymatic activity assays

Name	Structure	Rank
astragalin	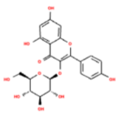	79.86
isoliquiritin apioside	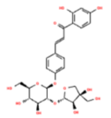	80
pectolinarin	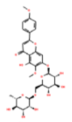	80.14
epimedin B	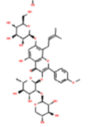	81.14
epimedin A	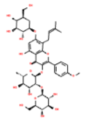	81.29
kaempferol 7-O-neohesperidoside	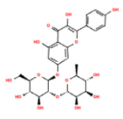	82.71

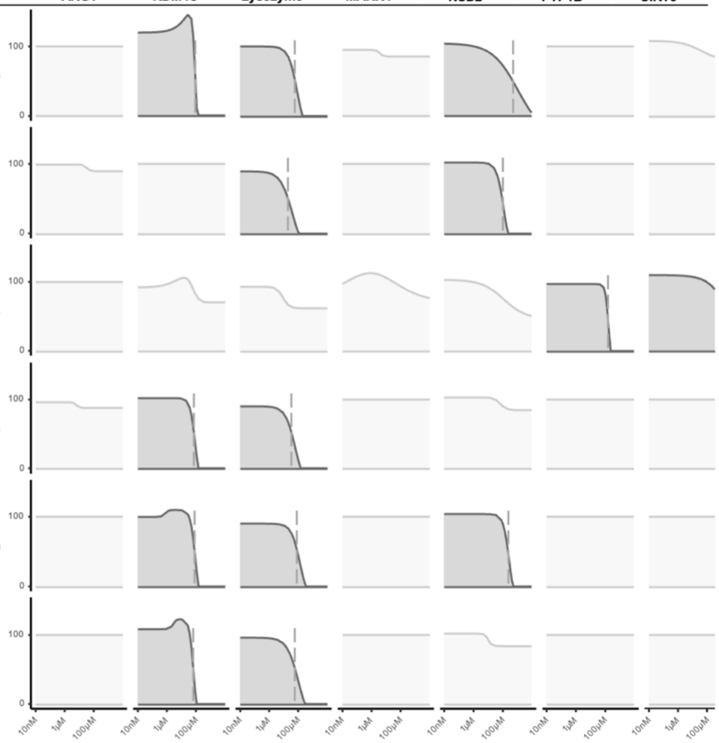

Extended Data Table. 2f.

Summary table of compound ranking and enzymatic activity assays

Name	Structure	Rank
oroxindin	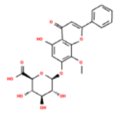	84
Apigenin 7-O-(2G-rhamnosyl) gentiobioside	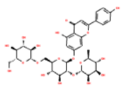	85
narcissin	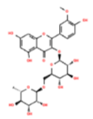	85.71
calendoflavoside	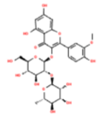	86.43
naringin	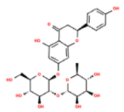	87.29
typhaneoside	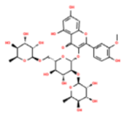	87.57

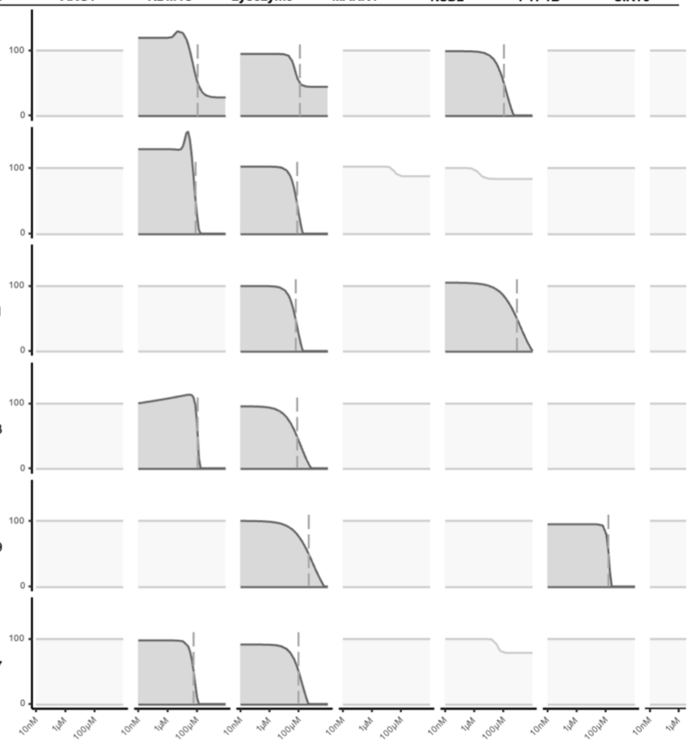

Extended Data Table. 2g.

Summary table of compound ranking and enzymatic activity assays

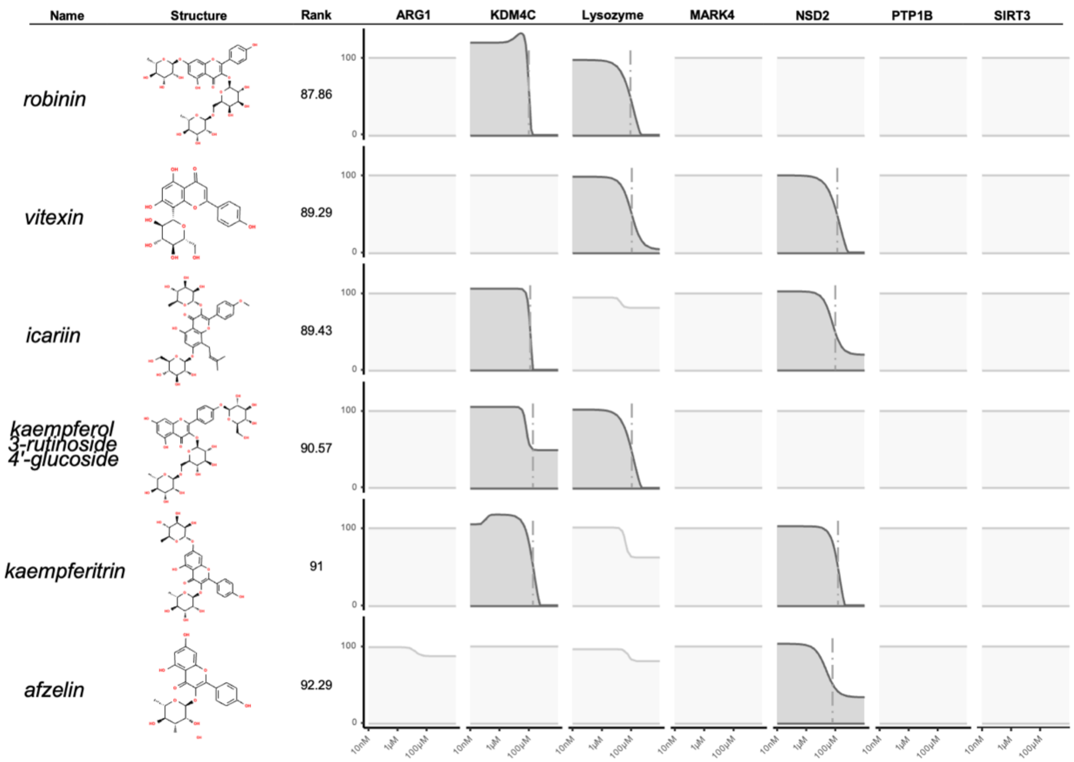

Extended Data Table. 2h.

Summary table of compound ranking and enzymatic activity assays

Name	Structure	Rank	ARG1	KDM4C	Lysozyme	MARK4	NSD2	PTP1B	SIRT3
Vitexin 2-O-rhamnoside	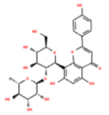	93							
epimedin C	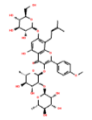	94							
populin	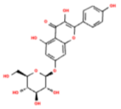	94							
Naringin dihydrochalcone	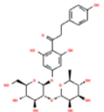	95.29							
nicotiflorin	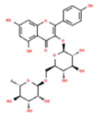	95.71							
kaempferol 3-gentiobioside	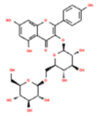	96.29							

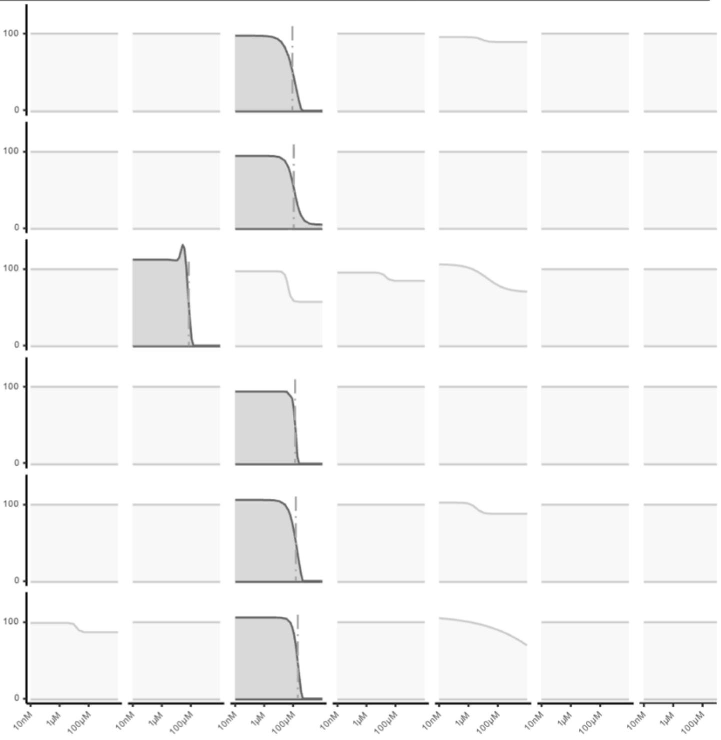

Extended Data Table. 2i.

Summary table of compound ranking and enzymatic activity assays

Name	Structure	Rank
chromen-2-one xylofuranoside	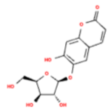	96.57
fluorescein-digalactoside	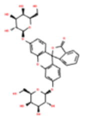	96.71
camelliaside	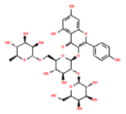	100
troxerutin	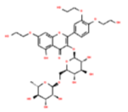	100

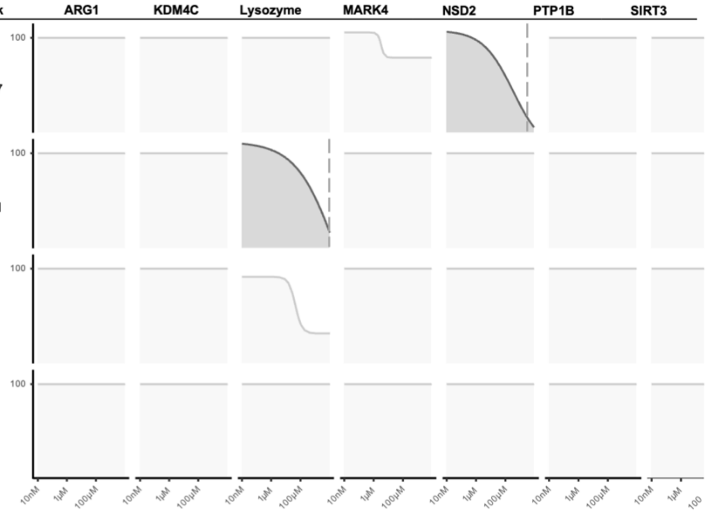

Extended Data Table. 2j.

Extended Data Table. 2: Dose-dependent inhibition of enzyme activity by a library of flavonoids.

a-j, Concentration-dependent inhibition observed for 58 flavonoids in enzyme activity assays for Arginase 1 (ARG1), Lysine demethylase 4C (KDM4C), Lysozyme, MAP/microtubule affinity-regulating kinase 4 (MARK4), Histone-Lysine N-methyltransferase (NSD2), Tyrosine-protein phosphatase non-receptor type 1 (PTP1B), and Siturin-3 (SIRT3). Compounds are sorted based on the decreased order of overall efficacy of enzyme inhibition; for the ranking methodology see the Materials and Methods section.

Extended Data Table. 3: Results of differential expression and pathway analysis of proteomics data. [See .xlsx file]

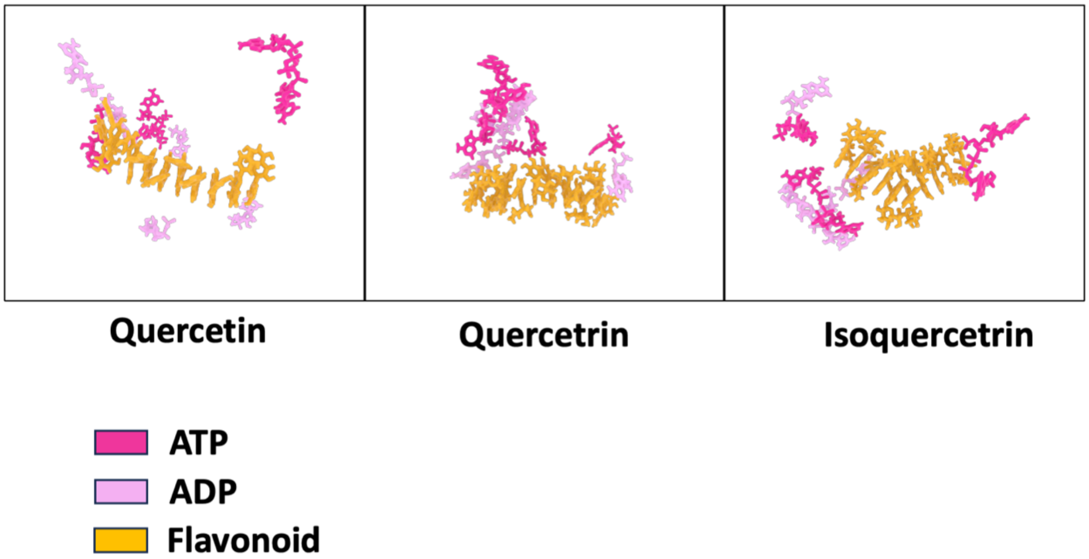

Extended Data Fig.1: Flavonoids simulated with ATP and ADP..

a, States taken after 100 ns of simulation of ten flavonoid molecules with five adenosine triphosphate (ATP) and five adenosine diphosphate (ADP) molecules. Videos of the 100 ns simulations are provided, and each video shows simulations in triplicate, s1, s2, s3, for quercetin, quercitrin and isoquercitrin respectively.

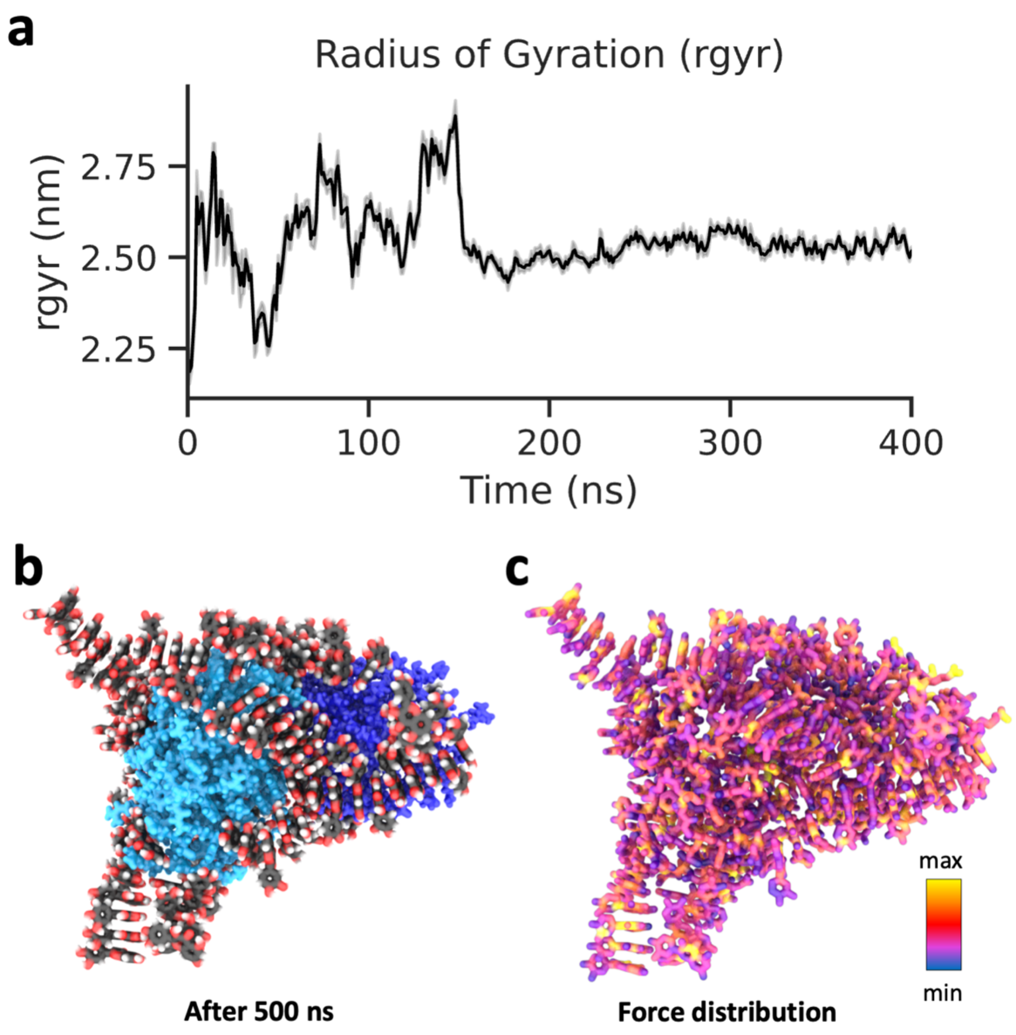

Extended Data Fig. 2: MDS reveals flavonoid supramolecular structures influence intermolecular mobility and mechanical force distribution.

a, Radius of gyration (RGYR) from MDS of two lysozyme molecules and 128 quercetin molecules. **b,** A single state from the end of the 400 ns MDS in **a**. **c**, The same state as **b** rendered to indicate the relative force experienced by each atom**.**

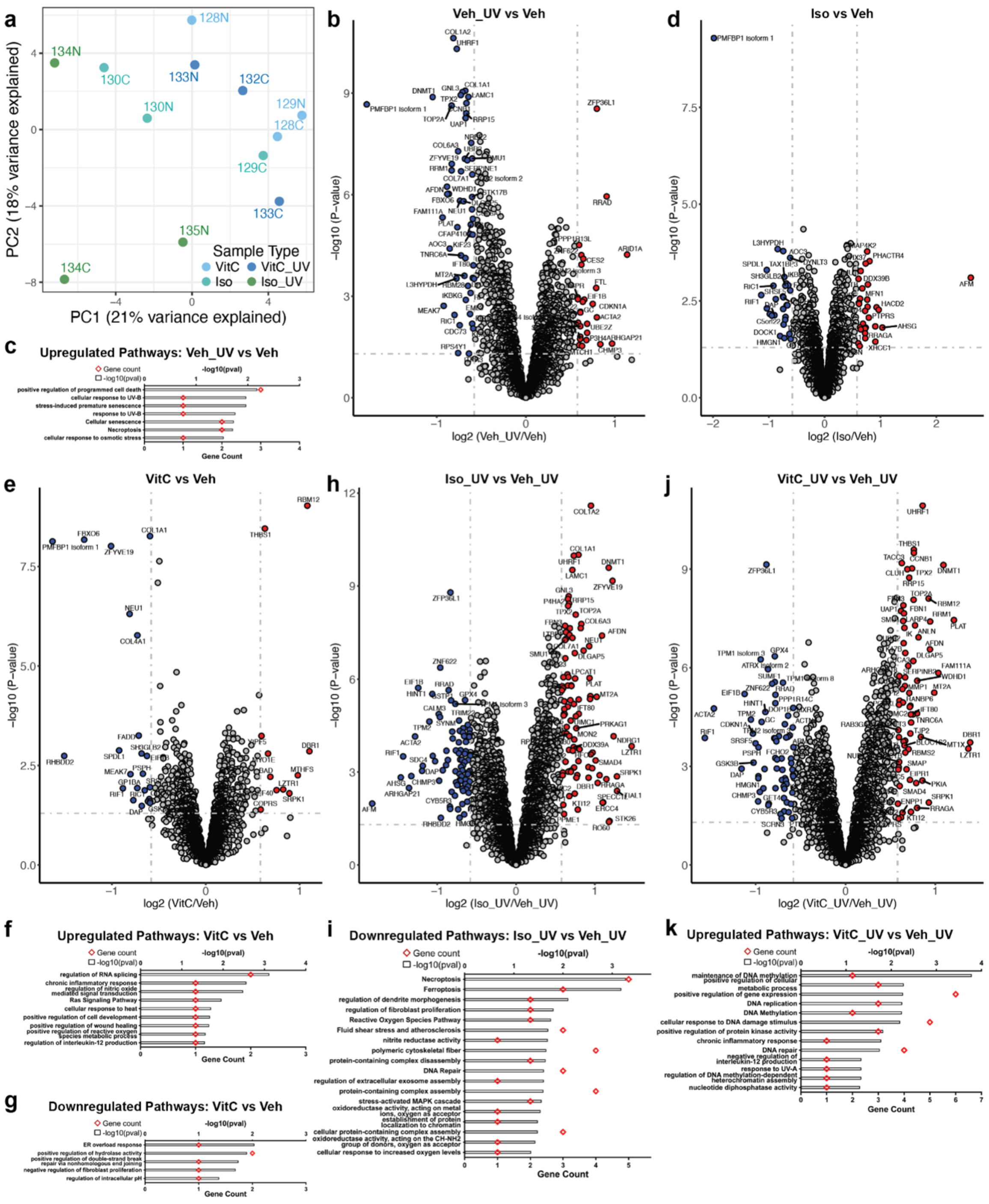

Extended Data Fig. 3

Extended Data Fig. 3: Proteomics data analyses suggests treatment of UV-exposed fibroblasts with isoquercitrin and vitamin C induces protection through distinct mechanisms and pathways.

a, Focused PCA plot showing clustering of only the proteomics samples treated with isoquercitrin or vitamin C, with and without UV-exposure. **b, d, e, h, j,** Volcano plots of proteins differentially expressed in Veh_UV vs Veh, Iso vs Veh, VitC vs Veh, Iso_UV vs Veh_UV and Vitc_UV vs Veh_UV proteomic profile comparisons, respectively. Grey dotted horizontal and vertical lines denote a p-value cutoff of 0.05 and log2fc cutoff of 0.58 (1.5-fold change), respectively. Proteins denoted by red and blue dots are significantly up- and downregulated, respectively. **c, f, k,** Pathways upregulated in Veh_UV vs Veh, VitC vs Veh, and VitC_UV vs Veh_UV proteomic profile comparisons, respectively. **g, i,** Pathways downregulated in VitC vs Veh and Iso_UV vs Veh_UV proteomic profile comparisons, respectively.
